# DeePhage: distinguishing virulent and temperate phage-derived sequences in metavirome data with a deep learning approach

**DOI:** 10.1093/gigascience/giab056

**Published:** 2021-09-08

**Authors:** Shufang Wu, Zhencheng Fang, Jie Tan, Mo Li, Chunhui Wang, Qian Guo, Congmin Xu, Xiaoqing Jiang, Huaiqiu Zhu

**Affiliations:** State Key Laboratory for Turbulence and Complex Systems and Department of Biomedical Engineering, College of Engineering, Peking University, Beijing 100871, Beijing, China; Center for Quantitative Biology, Peking University, Beijing 100871, Beijing, China; State Key Laboratory for Turbulence and Complex Systems and Department of Biomedical Engineering, College of Engineering, Peking University, Beijing 100871, Beijing, China; Center for Quantitative Biology, Peking University, Beijing 100871, Beijing, China; State Key Laboratory for Turbulence and Complex Systems and Department of Biomedical Engineering, College of Engineering, Peking University, Beijing 100871, Beijing, China; Center for Quantitative Biology, Peking University, Beijing 100871, Beijing, China; Peking University-Tsinghua University - National Institute of Biological Sciences (PTN) joint PhD program, School of Life Sciences, Peking University, Beijing 100871, Beijing, China; Peking University-Tsinghua University - National Institute of Biological Sciences (PTN) joint PhD program, School of Life Sciences, Peking University, Beijing 100871, Beijing, China; State Key Laboratory for Turbulence and Complex Systems and Department of Biomedical Engineering, College of Engineering, Peking University, Beijing 100871, Beijing, China; Center for Quantitative Biology, Peking University, Beijing 100871, Beijing, China; Department of Biomedical Engineering, Georgia Institute of Technology and Emory University, GA 30332, Atlanta, USA; State Key Laboratory for Turbulence and Complex Systems and Department of Biomedical Engineering, College of Engineering, Peking University, Beijing 100871, Beijing, China; Center for Quantitative Biology, Peking University, Beijing 100871, Beijing, China; Department of Biomedical Engineering, Georgia Institute of Technology and Emory University, GA 30332, Atlanta, USA; State Key Laboratory for Turbulence and Complex Systems and Department of Biomedical Engineering, College of Engineering, Peking University, Beijing 100871, Beijing, China; Center for Quantitative Biology, Peking University, Beijing 100871, Beijing, China; State Key Laboratory for Turbulence and Complex Systems and Department of Biomedical Engineering, College of Engineering, Peking University, Beijing 100871, Beijing, China; Center for Quantitative Biology, Peking University, Beijing 100871, Beijing, China; Department of Biomedical Engineering, Georgia Institute of Technology and Emory University, GA 30332, Atlanta, USA; Institute of Medical Technology, Peking University Health Science Center, Beijing 100191, Beijing, China

## Abstract

**Background:**

Prokaryotic viruses referred to as phages can be divided into virulent and temperate phages. Distinguishing virulent and temperate phage–derived sequences in metavirome data is important for elucidating their different roles in interactions with bacterial hosts and regulation of microbial communities. However, there is no experimental or computational approach to effectively classify their sequences in culture-independent metavirome. We present a new computational method, DeePhage, which can directly and rapidly judge each read or contig as a virulent or temperate phage–derived fragment.

**Findings:**

DeePhage uses a “one-hot” encoding form to represent DNA sequences in detail. Sequence signatures are detected via a convolutional neural network to obtain valuable local features. The accuracy of DeePhage on 5-fold cross-validation reaches as high as 89%, nearly 10% and 30% higher than that of 2 similar tools, PhagePred and PHACTS. On real metavirome, DeePhage correctly predicts the highest proportion of contigs when using BLAST as annotation, without apparent preferences. Besides, DeePhage reduces running time vs PhagePred and PHACTS by 245 and 810 times, respectively, under the same computational configuration. By direct detection of the temperate viral fragments from metagenome and metavirome, we furthermore propose a new strategy to explore phage transformations in the microbial community. The ability to detect such transformations provides us a new insight into the potential treatment for human disease.

**Conclusions:**

DeePhage is a novel tool developed to rapidly and efficiently identify 2 kinds of phage fragments especially for metagenomics analysis. DeePhage is freely available via http://cqb.pku.edu.cn/ZhuLab/DeePhage or https://github.com/shufangwu/DeePhage.

## Introduction

In a microbial community, phages are the major component of the viral genetic materials. It is estimated that the number of phages is on average 10 times higher than that of bacteria [1]. They may destroy bacteria but meanwhile in some situations benefit populations of bacteria, and thus crucially impact the composition of the microbial community [2]. With the development of high-throughput sequencing technology, a large number of novel phages have been discovered from metagenomes and viromes, in which viral particles are first enriched before sequencing [3,4]. However, the analysis of these phage sequences is a great challenge because the reference genomes of phages are very limited as a result of the fact that most phages cannot be cultured independently. There are far fewer complete phage genomes in current databases than those of bacteria; therefore there are a large number of sequences from virome data for which regions with homology to the known phages cannot be found [3]. In addition, unlike bacteria, phages lack the universal marker genes such as 16S ribosomal RNA [5], so many species identification strategies designed for bacterial analysis are not applicable to phages. Moreover, for mobile elements such as phages, sequence assembly is often poorer than for bacteria, usually because the mobile elements carry repetitive regions such as insertion sequences and share sequences that occurred among different genomes [6]. As a result, the large number of short fragments in metagenomic data also increases the difficulty of the analysis.

To overcome these difficulties, several computational tools focusing on 2 major tasks have been developed to analyse the phage sequences from metagenome or virome. One of the tasks is to identify phage fragments in metagenomic data, such as the tools VirSorter [7], VirFinder [8], MARVEL [9], virMine [10], and PPR-Meta [11]. Especially, we have developed PPR-Meta, a tool with high performance that demonstrates much better accuracy than the related tools. Another task is to assign the host for a given phage contig, which can be performed by such tools as WIsH [12], VirHostMatcher [13], and Hostphinder [14]. However, these tools cannot answer the question of how the newly discovered phages interact with their hosts. According to the interaction mode, which is also referred to as the phage lifestyle, phages can be divided into the virulent phages and the temperate phages [15]. When a virulent phage infects its host, it will produce many progeny as soon as the phage's DNA is injected into the host cell and then causes the death of the host through bacterial lysis [15]. In contrast, temperate phages can undergo the lysogenic cycle and lytic cycle. In the lysogenic cycle, a temperate phage will integrate its genome into the host chromosome, which is also referred to as a kind of prophage, and then copies its genome together with the host chromosome [1]. When induced by appropriate conditions, especially nutritional conditions and a sufficient number of co-infecting phages, temperate phages will enter the lytic cycle, followed by releasing the viral particle and killing the host through bacterial lysis [16]. Such different processes have a significant influence on the microbiota especially in the human gut, which could be highly correlated with human diseases or the treatment of human disease. Although some kinds of hot spots, such as phage therapies that make use of virulent phages in the context of therapeutic applications [17], have been investigated, limited by current bioinformatics tools, comparatively little is known about these different lifecycles in view of their prevalence in the human gut [18]. Therefore, it is important to distinguish virulent and temperate phages for further understanding of phage-host interactions.

Although the classification strategy of this issue for virome data is still a challenge, there are several noteworthy works that help to characterize the virulent and temperate phages. Even in phages lacking marker genes, those studies show that they may have some functional genes, which are high-frequency genes and can tell us whether a given phage is virulent or temperate in a relatively credible way. For example, Emerson et al. found that there were some functional genes for temperate phages, such as integrase and excisionase [19]; Schmidt et al. found that leucine substitution in the DNA polymerase (*pol*A) gene had a strong connection with temperate phages [20]. Notably, McNair et al. designed a computational tool called PHACTS to identify whether a phage with a complete or partial proteome is virulent or temperate [15]. This tool uses all the sequence information of proteins from a phage genome and uses the random forest as a classifier to make the judgment. Researchers further found that the existence of some kinds of genes helped PHACTS present good results. For example, virulent phages usually have genes related to phage lysis, nucleotide metabolism, or structural proteins, while temperate phages usually contain genes related to toxins, excision, integration, lysogeny, or regulation of expression [15]. Unfortunately, such strategies may not apply to metagenomic data. To date, it is still a difficult task to reconstruct complete genomes of all organisms in the metagenomic data. Therefore, only a few DNA fragments may contain those functional genes that can help to make the judgment. According to the report of PHACTS, this tool can achieve accuracy >95% if ≥25 proteins are provided from a phage genome. However, if fewer proteins are obtained, the accuracy of PHACTS decreases. When only 5 proteins are obtained from a phage, PHACTS only achieves an accuracy of ∼65%; if only 2 proteins are obtained from a phage, PHACTS seems to produce random results, with an accuracy <55%. Considering that most of the DNA sequences in metagenomic data are short fragments that only contain a few genes or even incomplete genes, it is essential to develop a tool that does not depend on information from sufficient proteins to reach the level of functional genes, while making the judgment directly for each short DNA fragment in metagenomic data. Recently, a new tool, PhagePred, was developed to identify phages’ lifestyle in metagenomic sequences [21]. Based on the Markov model, PhagePred uses *k*-mer frequencies as the sequence feature to test the dissimilarity measures between new contig and 2 kinds of phages. Then it is used to determine the new contig's lifestyle. PhagePred was tested on various contig lengths (500, 1,000, 3,000, 5,000, and 10,000 bp). However, as a global statistic, such *k*-mer methods will generate much noise with a short metagenomic sequence. When PhagePred is applied to sequences from 500 to 10,000 bp, for a short sequence, the *k*-mer frequencies may be different from long sequences, and the variance may be higher than long sequences. Thus, the *k*-mer–based method may not be applicable for all ranges of contig length for PhagePred. Another concern is that *k*-mer frequency will lose detailed sequence information when encoding sequence to *k*-mer feature vectors. Considering all the aforementioned shortcomings, it is better to develop a new method that is more suitable for short phage sequences.

In this article, we present a 2-class classifier, DeePhage, to identify whether a DNA fragment is derived from a virulent phage or a temperate phage. Using the information of every nucleotide without manually extracted features, DeePhage encodes sequences in “one-hot” form. Such representations are suitable for the convolutional neural network (CNN) model to detect helpful motifs for classification, which are commonly used in biological sequence identification. Together with other kinds of neural network layers, DeePhage learns to recognize different features between virulent and temperate sequences and then outputs a score indicating the possibility of being a certain kind of phage sequence. Tested on the same data, DeePhage significantly outperforms the related methods PhagePred and PHACTS on the metric of computational efficiency, using only 1/245 and 1/810 the computation time, respectively. Simulation tests on 5-fold validation show that DeePhage outperforms these 2 methods in accuracy metrics by ∼10% and 30%. DeePhage's evaluations on real metavirome data of bovine rumen are better than PhagePred and PHACTS with much more accurate results, which use annotations of the BLAST method as a relatively accurate reference. Meanwhile, we present a new strategy to conveniently detect the phage transformation by tracing specific types of phage contigs, which can explore the influence of phages that contribute to microbial communities and even to human diseases. DeePhage can be used to analyse virome data and metagenomic data directly. While handling metagenomic data, users need to first identify the phage sequences using related software, such as PPR-Meta [11] as mentioned above, and then use DeePhage to further annotate the phage sequences.

## Material and Methods

### Data construction

Considering that there exist no real virome data with reliable lifestyle annotations for each sequence to use as a benchmark, we constructed artificial contigs extracted from well-annotated complete phage genomes as the benchmark to train and test the algorithm. We downloaded 227 complete phage genomes with lifestyle annotations from the dataset of McNair et al., including 79 virulent phages and 148 temperate phages [15]. Among these phages, we removed 2 virulent phages from the dataset: mycobacteriophage D29 (accession: NC_001900) and lactococcus lactis bacteriophage ul36 (accession: NC_004066), because the lifestyle of these 2 phages may be ambiguous. Although these 2 phages are annotated as virulent phages, researchers found that they both contained functional integrases, indicating that they can integrate their genomes into host chromosomes like temperate phages [15]. Besides, D29 is very similar to the temperate phage L5 [22], while ul36 has 46.6% homology with the temperate phage Tuc2009 [23]. Therefore, 77 virulent phages and 148 temperate ones are used in the dataset of the present study, named Dataset-1. In addition, following the phage lifestyle dataset construction strategy of Song [21], we recruited >1,500 phage RefSeq genomes from the NCBI database [24]. Their lifestyle annotations are labelled using a bioinformatic method [25]. Excluding the overlapped phages in Dataset-1, we included 1,211 virulent and 429 temperate phage genomes (Dataset-2). Dataset-1 is manually curated with credible lifestyle annotations, while Dataset-2 is not. Therefore, based on genomes, we used all phages of Dataset-2 and 80% of the phages of Dataset-1 to be the training set, and 20% of the phages of Dataset-1 to be the test set. Detailed information on each phage genome and their host information from Dataset-1 and Dataset-2 is provided in Supplementary Data 1. Moreover, a usage label (Training usage or Test usage) indicates in which cross-validation the phage genome was used for training or test set in Supplementary Data 1. For convenience, herein the virulent phages are referred to as the positive sample and the temperate phages as the negative sample.

We further used MetaSim (v0.9.1) [26] to extract artificial contigs from the complete phage genomes. Considering that the length of contigs in real metagenomes may cover a wide range, we divided the artificial contigs into 4 groups A–D, according to their length range, 100–400, 400–800, 800–1,200, and 1,200–1,800 bp, respectively. Those 4 groups may cover the length of raw reads and the mean length of assembled contigs from the next-generation sequencing technology. We evaluated the performance of DeePhage on the 4 different groups.

We also used real virome data to estimate the reliability of DeePhage qualitatively. We downloaded virome data of bodily fluids in the bovine rumen [27] from MG-RAST [28]. They were downloaded as raw reads (accessions: mgm4534202.3 and mgm4534203.3). We used SPAdes (v3.13.0) [29] to assemble the raw reads and obtained 118,918 contigs with an N50 of 291 bp.

### Mathematical model of phage sequences

To evaluate the feasibility of the sequence signature used for classifying virulent and temperate phages, we first analysed the distribution of *k*-mer frequencies, which have been widely used to distinguish genomes from different species, among virulent phage genomes and temperate phage genomes from Dataset-1. Using 4-mer frequencies with the anosim (analysis of similarities) test shows that there is a significant difference between those 2 groups (*R* = 0.080, *P* = 0.001), which means that they have different sequence signatures to characterize the 2 categories of phage genomes. To visualize this result, we performed principal component analysis (PCA) [30] based on 4-mer frequencies features (see Supplementary Fig. S1).

Although *k*-mer frequencies have shown their ability to classify virulent and temperate phage genomes, using such frequencies to characterize short DNA fragments will usually be hindered by noise [12]. Also, because global statistics may miss some local information, it is difficult to use *k*-mer frequencies to characterize the details of mobile elements that contain a mosaic structure [31]. To describe the local sequence information in detail, we consider the one-hot encoding form, which can represent every base continuously and entirely. For each sequence, we used the “one-hot” encoding form to represent each base in a sequence. Specifically, bases A, C, G, and T were represented by [0, 0, 0, 1], [0, 0, 1, 0], [0, 1, 0, 0], and [1, 0, 0, 0], respectively. In particular, the one-hot encoding form could be regarded as a special 1-mer frequency detector.

### Algorithm structure of DeePhage

Deep learning algorithms are recognized as an extremely effective method in many fields including in biology. Compared with recurrent neural networks, the CNN models are faster to train and more efficient in sequential spatial correlations [32]. Specifically, CNN is a universal network for extracting local patterns in terms of biology, which in the present context can be used as a motif detector of DNA sequences. In DeePhage, we present a deep learning algorithm with CNN models to handle the input sequences represented by the one-hot encoding form. The network contains 8 layers: a 1D convolutional (Conv1D) layer, a 1D maximum pooling (Maxpooling) layer, a 1D global average pooling (Globalpooling) layer, 2 batch normalization (BN1 and BN2) layers, a dropout (Dropout) layer, and 2 dense (Dense1 and Dense2) layers.

The Conv1D layer takes a sequence encoded by an ${L} \times 4$ matrix $\mathbf{X}$ (*L* is the length of the sequences, equal to 400, 800, 1,200, and 1,800) as the input and generates a total of *F* feature maps as output by a corresponding *F* convolutional kernels. Those kernels can be used to detect vital motifs. Using ReLU (Rectified Linear Unit) [33] as the activation function, the Conv1D layer outputs an ${L} \times {F}$ matrix ${\mathrm{Y}^C}$ and computes for the *f*th feature map at the *l*th location like this:

\begin{equation*} \mathrm{Y}_{{l,f}}^C = {\mathrm{ReLU}}\left(\,\,\mathop \sum \nolimits_{m\,\,{\mathrm{ = \,\,0}}}^{{\mathrm{M - 1}}} \mathop \sum \nolimits_{n\,\,{\mathrm{ = \,\,0}}}^3 W_{m,n}^{\mathrm{f}}{X_{{\mathrm{l + }}m,n}} + b_{f}^{\mathrm{C}}\right), \end{equation*}

 

\begin{equation*} {\mathrm{for}}\,\,l\,\, = 0,1,2, \ldots ,{L} - 1,\,\,f\,\, = \,\,0,1,2, \ldots ,{F} - 1. \end{equation*}

The ${W^{\mathrm{f}}}$and ${{b}}_{f}^{\mathrm{C}}$are an M$\times $4 weight matrix and a bias of the *f*th kernel. The aforementioned ReLU function is defined as [33]:

\begin{equation*} {\mathrm{ReLU}}({x}) = \Bigl\{ \begin{array}{@{}*{1}{c}@{}} {{x\,\,}\,\,{\mathrm{if}}\,\,{x} \ge 0}\\ {0\,\,{\mathrm{if}}\,\,{x} < 0} \end{array}\,\,. \end{equation*}

As a traditional nonlinear function, the ReLU function is easier to train and achieves better performance, which can rectify the shortcomings of sigmoid functions. Those kernels scan sequences one after another to extract the features relevant to the classification, and the ReLU function achieves a nonlinear transformation.

Such a combination is followed by the Maxpooling layer downsampling the input representation by taking the maximum value over an input channel with a pooling size S1 and a stride size S2. The window is shifted along with each channel independently and can generate *F* new channels with the size of ${L^{\prime}}\,\,( {{\,\,L^{\prime}} = {L}/{\mathrm{S}}2{\mathrm{\,\,}}} )$. The Maxpooling layer outputs an ${L^{\prime}} \times \,\,{F}$ feature matrix ${\mathbf{Y}^M}$ and one of the pooling operation for a specific channel at the *l*th location is defined as follows:

\begin{equation*} Y_{{l,f}}^{\mathrm{M}} = \max \left(\,\,Y_{{l} \times {\mathrm{S2},f}}^{\mathrm{C}},Y_{{l} \times {\mathrm{S2 + 1},f}}^{\mathrm{C}},Y_{{l} \times {\mathrm{S2 + 2},f}}^{\mathrm{C}}, \ldots ,Y_{{l} \times {\mathrm{S2 + S1 - 1},f}}^{\mathrm{C}}\right), \end{equation*}

 

\begin{equation*} {\mathrm{for}}\,\,l\,\, = 0,1,2, \ldots ,{L^{\prime}} - 1,\,\,f\,\, = \,\,0,1,2, \ldots ,{F} - 1. \end{equation*}

Its main function is to reduce the dimensions of each input channel using the final summarized features, which can also adapt to location variations of valuable features.

Features from the Maxpooling layer are passed to the BN1 layer to scale the inputs. At each batch, it usually transforms inputs to have a mean close to 0 and a standard deviation close to 1, which can avoid the vanishing gradient problem and accelerate the convergence rate of the model. Thus, the output feature matrix ${\mathbf{Y}^{{\mathrm{B1}}}}$ of the BN1 layer is also an ${L^{\prime}} \times \,\,{F}$ matrix as ${\mathbf{Y}^{\mathrm{M}}}$ but is scaled.

The next is a Dropout layer, which randomly drops a certain proportion (denoted as *P*) of input elements by setting them to zero during training [32]. The output ${{\mathbf{Y}}^{{\mathrm{Db}}}}$ is formulated as:

\begin{equation*} {{\bf{Y}}^{{\mathrm{Dp}}}} = {\bf{K}} \odot {{\bf{Y}}^{{\mathrm{B}}1}},\,{\mathrm{where}}\,{\bf{K}} \sim B(1,{P}). \end{equation*}

The drop mask ${\mathbf{K}}$ denotes a Bernoulli distribution with *n* = 1 and *p* = *P*. It could effectively reduce overfitting especially in our small dataset [34].

After a dropout layer, the Globalpooling layer takes the ${{\mathbf{Y}}^{{\mathrm{Db}}}}$ as input and reduce features from the same channel into 1 dimension by using the average value of those features, which can integrate global spatial information. More formally:

\begin{equation*} y_{f}^{\mathrm{G}} = \frac{1}{{{L^{\prime}}}}\,\,\mathop \sum \nolimits_{l\,\, = \,\,0}^{{L^{\prime}} - 1} Y_{l,{f}}^{{\mathrm{Dp}}},\,\,{\mathrm{for}}\,\,\,\,f = \,\,0,1,2, \ldots ,{F} - 1, \end{equation*}

where $y_{f}^\mathrm{G}$ is the average value of features from the *f*th input channel. Considering all the *F* channels from the previous layer, the output of the Globalpooling layer **y**^G^ is an *F*-dimensional vector.

Subsequently, a Dense1 layer using the ReLU function as activation function outputs *R* units. It has an *R* × *F* weight matrix ${\mathbf{W}^{{\mathrm{D1}}}}$ and an *R*-dimensional bias vector ${b^{{\mathrm{D1}}}}$. Each output units is processed by:

\begin{equation*} y_{r}^{{\mathrm{D}}1} = {\mathrm{ReLu}}\left( {\mathop \sum \nolimits_{{f\,\,} = \,\,0}^{{F} - 1} W_{{r},{f}}^{{\mathrm{D}}1}y_{f}^{\mathrm{G}} + b_{r}^{{\mathrm{D}}1}} \right),{\mathrm{\,\,for}}\,\,\,\,r = \,\,0,1,2, \ldots ,{R} - 1. \end{equation*}

The Dense1 layer can compile the features from different input channels together and finally generate an *R*-dimensional vector ${\mathbf{y}^{{\mathrm{D1}}}}$, while the Conv1D layer just extracts features into different feature maps.

The vector ${\mathbf{y}^{{\mathrm{D1}}}}$ is then sent into a BN2 layer to generate a new feature vector ${\mathbf{y}^{{\mathrm{B2}}}}$ that has a mean close to 0 and a standard deviation close to 1, which has the same effect as the BN1 layer.

Using a sigmoid function as an activation function, the final layer is the Dense2 layer and outputs only 1 score between 0 and 1 representing the probability of prediction. Using an *R*-dimensional weight vector ${\mathbf{W}^{{\mathrm{D2}}}}$ and a bias scalar ${b^{{\mathrm{D2}}}}$, the output score is given by:

\begin{equation*} {y^{{\mathrm{D}}2}} = {\mathrm{sigmoid}}\left( {\mathop \sum \nolimits_{r\,\, = \,\,0}^{{\mathrm{R}} - 1} W_r^{{\mathrm{D}}2}y_r^{{\mathrm{B}}2} + {b^{{\mathrm{D}}2}}} \right). \end{equation*}

The sigmoid function is defined as:

\begin{equation*} {\mathrm{sigmoid\,\,}}\left( {x} \right) = \frac{1}{{1 + {{e}^{ - {x}}}}}\,\,. \end{equation*}

**Figure 1:**
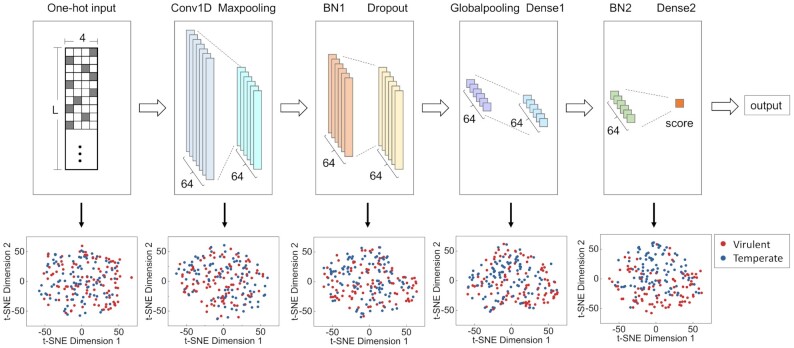
Structure of deep learning neural network and visualization of 5 layers by reducing dimensions. DeePhage uses the CNN model as the classifier. The neural network (in the upper part) takes the sequence in the “one-hot” coding form as input and outputs a score between 0 and 1. In general, the sequence with a score >0.5 can be referred to as the virulent phage–derived fragment and the sequence with a score <0.5 can be referred to as the temperate phage–derived fragment. The visualization demonstrates the learning process of DeePhage. The performance would be better when we observe a deeper layer (in the lower part).

By default, a sequence with a score >0.5 would be regarded as a positive sample (a virulent phage) and a sequence with a score <0.5 would be regarded as a negative sample (a temperate phage). When training, we used the Adam optimizer [35] (learning rate = 0.0001), binary cross-entropy as the loss function, and 32 as the batch size to train the neural network and update network weights. Altogether, we found that setting the size *F* to 64, *M* to 6, S1 to 3, S2 to 3, *P* to 0.3, and *R* to 64 produced the best performance. The structure of DeePhage is shown in the upper part of Fig. 1.

It is worthwhile to know more about the importance of the encoding method for sequences and each specific layer in our model, so we tested 6 different models (including DeePhage) by using *k*-mer frequencies as an encoding representation or removing a certain layer. The 6 model architectures (DeePhage, Kmer-4, No-Maxpooling, No-Dropout, No-Globalpooling, and No-BN) are shown in Supplementary Fig. S2 and their performance is shown in Supplementary Table S1. It can be seen that the Kmer-4 model produced a terrible prediction. As mentioned above, when we used 4-mer frequencies to characterize each phage at the level of genome sequences, it could slightly distinguish 2 kinds of phages. It was proved that *k*-mer frequencies did not have enough power to represent short sequences and are fit only for capturing the global signature of long sequences rather than the local signature of short sequences. Thus, we used convolutional kernels to detect sequence motifs, where each column of kernels represents the probabilities of having A/C/G/T at 1 position (position weight matrices) [36]. Those kernels can involve *k*-mer frequencies but in a more detailed and local way. As for those models removing a certain layer, the performance decreased compared with DeePhage. Especially, the prediction accuracy decreased 14% and 5% when using a model without a Globalpooling layer and BN layers (No-Globalpooling and No-BN model), respectively. Other models decreased slightly. We can see that the architecture and the one-hot encoding representation of DeePhage are better than others.

Although deep neural networks are considered to be black-box models, we hope to gain insights into the learning process for features. We chose 5 layers (One-hot input, Conv1D, BN1, Globalpooling, and BN2) to observe their learned features. Because it is hard to gain an intuitive display about high-dimension features, we used t-distributed stochastic neighbour embedding (t-SNE) [37], which is a machine learning algorithm for dimensionality reduction, for the visualization of high-dimensional data in a 2D projected space. After the training process, we first used PCA to reduce the features of the 5 aforementioned layers into a 10D space and then used t-SNE to reduce them into a 2D space using the sequences from Group D. The visualizations of the 5 layers are shown in the lower part of Fig. 1. It could be seen that the effects of classification are better when focusing on the deeper layers. In detail, 2 types of phages were first mixed together and then separated gradually, which demonstrated the learning process of DeePhage. Furthermore, it should be emphasized that the visualizations by dimensionality reduction cannot reflect the complete power of DeePhage.

Considering other lengths of sequences beyond our 4 trained groups, we designed some strategies. For those sequences longer than 1,800 bp, DeePhage will split the sequence into several 1800-bp-long subsequences without overlapping, usually except the last subsequence. DeePhage will then use the neural network in the corresponding group to predict each subsequence, and calculate the weighted average score according to the score and length of each subsequence. Because training the neural network using long sequences is very time-consuming, we do not train additional neural networks for longer sequences. For those sequences shorter than 100 bp, DeePhage uses the neural network in Group A to make a prediction.

## Results

### Identification performance of DeePhage, PhagePred, and PHACTS

We first used the 5-fold cross-validation to evaluate the performance of DeePhage. To test whether DeePhage can distinguish the lifestyle of novel phages, for each validation, we divided the training set and the test set on the basis of complete genomes rather than artificial contigs and then simulated 80,000 training sequences and 20,000 test ones using MetaSim [26]. The performance evaluation criteria here are defined as follows: Sn = TP/(TP + FN); Sp = TN/(TN + FP); and Acc = (TP + TN)/(TP + TN + FN + FP). Among these criteria, sensitivity (Sn) and specificity (Sp) are used to evaluate the accuracy of virulent phages and temperate phages, respectively, while accuracy (Acc) is used to evaluate the overall performance. As shown in Table 1, DeePhage demonstrates overall reliable and stable performance with Acc from 76% to 89%. Compared with PhagePred, the Acc criteria of PhagePred are ∼11–14% lower than DeePhage, as shown in Table 1. We used $d_{2}^{*}$ dissimilarity measures, *k*-mer length of 9, and Markov order of 2 as prediction parameters. Although the Sn values are close to those of DeePhage, the Sp values are >18% lower than DeePhage. This result is probably owing to some aforementioned limitations when the *k*-mer–based method is applied to short sequences. Thus, the performance of DeePhage is superior to that of PhagePred. Compared with PHACTS, the Acc values of PHACTS are only ∼50%, which is also lower than DeePhage. For PHACTS, sequences are input in the form of amino acid sequences and sequences without coding region are flagged as wrong predictions. Such results indicate that the input of functional genes with several proteins is not required for DeePhage. Therefore, our DeePhage method shows an evident advantage compared with the tool PHACTS. Because DeePhage can identify each DNA fragment as either virulent phage–derived sequence or temperate phage–derived sequence directly and independently, it would be a more suitable tool for analysing phages in metagenomic data. In this case, it is difficult to reconstruct the complete or near-complete genomes for phages from the data, especially for those with low abundance or in a low-coverage sequencing condition. Clearly, our tool DeePhage has the advantage of being applicable to processing the data by means of current short-read sequencing technologies and performs better when the short reads could be assembled into longer contigs. More details about the performance of the ROC curves and AUC scores of DeePhage in each rotation of the 5-fold cross-validation are shown in Supplementary Fig. S3.

**Table 1:** Results of 5-fold cross-validation for DeePhage, PhagePred, and PHACTS

		Mean ± SD			
Tool	Criterion (%)	Group A (100–400 bp)	Group B (400–800 bp)	Group C (800–1,200 bp)	Group D (1,200–1,800 bp)
DeePhage	Sn	77.3 ± 4.2	82.2 ± 3.4	86.2 ± 3.2	87.5 ± 3.3
	Sp	74.6 ± 6.9	84.4 ± 8.2	86.3 ± 10.2	89.5 ± 8.5
	Acc	76.2 ± 1.9	83.7 ± 2.5	86.6 ± 3.6	88.9 ± 2.9
PhagePred	Sn	75.8 ± 1.4	80.8 ± 1.9	84.2 ± 2.1	87.4 ± 2.0
	Sp	56.6 ± 5.0	60.3 ± 6.2	62.8 ± 7.2	64.7 ± 7.8
	Acc	65.7 ± 2.4	70.1 ± 2.9	72.9 ± 3.5	75.5 ± 3.6
PHACTS	Sn	73.7 ± 0.7	64.7 ± 1.0	65.8 ± 1.6	69.1 ± 1.7
	Sp	26.3 ± 1.5	36.9 ± 1.2	39.1 ± 0.3	42.3 ± 2.4
	Acc	48.6 ± 1.7	49.9 ± 1.2	51.5 ± 0.8	54.8 ± 1.5

The validation of each group was performed independently.

In general, sequences with scores near 0.5 are not as reliable as those sequences with a score near 0 or 1. Therefore, DeePhage is designed with an adjustable cut-off to filter out these uncertain predictions. Users can specify a cut-off using a parameter. In this way, a sequence with a score in the range (0.5 − cut-off/2, 0.5 + cut-off/2) will be labelled as “uncertain.” In general, with a higher cut-off, the percentage of uncertain predictions will be higher while the remaining predictions will be more reliable. We recommend 0.5 as a suitable value of cut-off. It means a sequence with a score in the range (0.25, 0.75) should be ignored. When tested on the cross-validation set, it can receive a high average AUC score and retain enough sequences. If hoping for a more reliable prediction, users could assign the parameter *t* to 0.50 as described in the manual of DeePhage. When 0.50 is used as cut-off, the main results do not change for analysing a real dataset. Thus, we do not use a cut-off in the following analysis.

### Comparison with PhagePred and PHACTS for protein sequence identification

**Figure 2:**
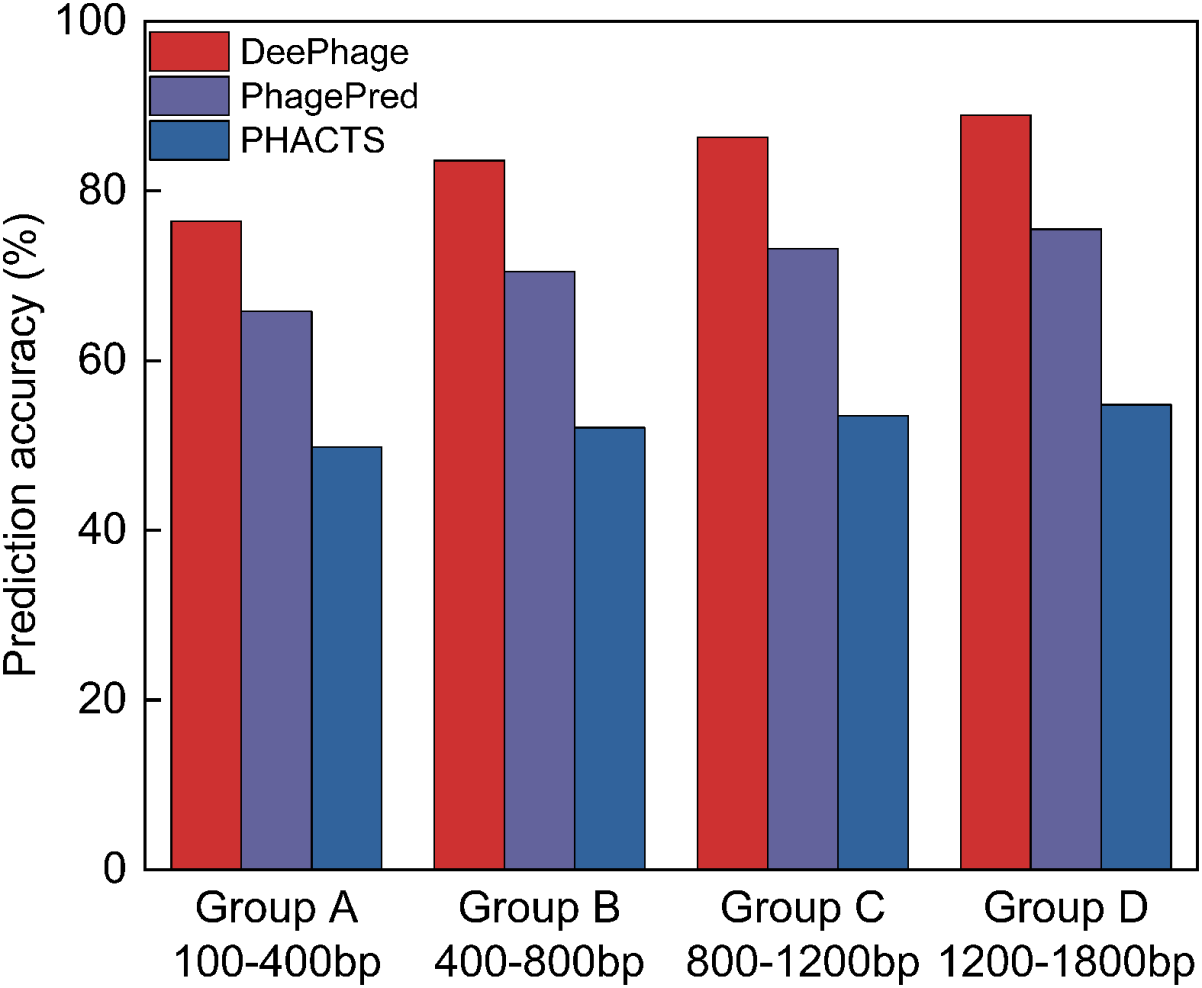
Comparison results of DeePhage, PhagePred, and PHACTS in each length group.

It should be noted that DeePhage and PHACTS are designed for different tasks: PHACTS is designed for complete genomes while DeePhage is designed for metagenomic fragments. Therefore, their input data requirements are actually different. PHACTS requires users to input all proteins (amino acid sequences) within 1 phage genome, so proteins from different phages should not be put into the same file. In contrast, DeePhage's requirement is only to input all DNA fragments (nucleic acid sequences), no matter whether they contain coding regions and whether they are from the same phage, and DeePhage can directly judge each fragment independently. Although it was difficult to compare 3 tools based on the same condition, we tried to test the performance of PHACTS only in DNA short fragments with coding region. Because PHACTS requires a collection of protein sequences as input, we first annotated the protein sequences of 100,000 DNA sequences of the test set in each length group using FragGeneScan (v1.31) [38], and proteins from the same sequence (sequences without coding regions were ignored) were input into the program PHACTS (v0.3). As for comparison, DeePhage and PhagePred are also used to predict these DNA sequences with coding regions. The total Acc (the number of correct predictions divided by the total number of sequences having the coding regions in each length group) of DeePhage, PhagePred, and PHACTS in each length group is shown in Fig. 2. For short fragments covering data sets of Group A–D, PHACTS demonstrates Acc values of ∼50%, which are nearly the results of random predictions. In Group D, PhagePred demonstrates Acc ∼76% and still 13% lower than that of DeePhage. In contrast, DeePhage can satisfactorily classify the sequences with Acc ∼76%–89%.

In addition, we have evaluated the performance of DeePhage, PhagePred, and PHACTS on the coding sequences (CDSs) from all 225 phage genomes. Because PHACTS could only process protein sequences, we extracted all CDSs from the genomes according to the GenBank annotation and each CDS was independently input to PHACTS (in the form of amino acid sequences), PhagePred (in the form of nucleic acid sequences), and DeePhage (in the form of nucleic acid sequences). We found that PHACTS can only achieve an Acc of 54.3%, which is also near to random judgment results, while DeePhage achieves an Acc of 82.7%, nearly 30% higher than that of PHACTS. PhagePred achieves an Acc of 68.4%, which is also lower than DeePhage. Considering that the number of CDSs in each metagenomic fragment is limited, PHACTS actually has a limited ability to analyse metagenomic data especially when the complete genomes could not be reconstructed using these fragments. Overall, as a state-of-the-art tool designed for phage lifestyle classification from metagenomic data, DeePhage, a *de novo* tool using the deep learning algorithm, presents an efficient prediction method.

Also, DeePhage can handle large-scale high-throughput data within an acceptable running time. To test, we recorded the runtime of DeePhage, PhagePred, and PHACTS to predict 100 DNA sequences (converted to protein sequences for PHACTS) ranging from 100 to 1,800 bp. DeePhage spends 10 seconds, which is 245 times faster than PhagePred and 810 times faster than PHACTS (using 41 minutes and 135 minutes), when tested on a virtual machine with the following configuration: CPU: Intel Core i7 4790; and memory: 8G, DDR3. As for PHACTS, every sequence needs to be aligned and every prediction needs to be replicated 10 times, while DeePhage could directly predict every sequence without any alignments. Therefore, DeePhage is much faster than PHACTS.

### Evaluation of DeePhage, PhagePred, and PHACTS using real metavirome data

Although it was difficult to make exact evaluations using real data, some functional genes could help us to make an approximately effective assessment of our model. In this subsection, we used DeePhage to predict all the sequences in metavirome data of bovine rumen [27] with 118,918 contigs assembled by SPAdes (v3.13.0) [29]. As a result, 53.6% (63,691/118,918) of the contigs are predicted as virulent phage–derived contigs and 46.4% (55,227/118,918) as temperate phage–derived contigs. For assessment of DeePhage's prediction, we then assessed the RefSeq viral protein database [39] as a reference. Because the viral proteins labelled as “excision," “integration," or “lysogeny" are more likely to exist in temperate phages [15], we used those proteins to build an MTPD (mini temperate phage-derived) set containing 107 protein sequences. We then searched all 118,918 contigs against the MTPD data using Blastx v2.7.1 [40] and obtained 16 targeted contigs having homologous regions (e-value ≤ 1e−10, hits length ≥ 400). These hits present an extremely small proportion (16/118,918), which confirms that a huge quantity of data have no reliable homologous regions in known databases. When it comes to DeePhage, 13 of 16 targeted contigs can be identified as temperate phage–derived contigs, while only 6 and 10 contigs can be classified as temperate phage–like contigs by PhagePred and by PHACTS, respectively. It shows that DeePhage performs better than PhagePred and PHACTS and has rather the potential to analyse newly sequenced phage data. However, the fact that the prediction scores are nearly 0.5 shows that PHACTS actually made random inferences, while DeePhage had a majority of reliable scores and made better predictions. The information of 16 targeted contigs and predicted results by DeePhage, PhagePred, and PHACTS are listed in Table 2.

**Table 2:** Information of 16 targeted contigs and predicted results by DeePhage, PhagePred, and PHACTS

	Contig length				DeePhage		PHACTS		PhagePred
Contig ID	(bp)	Identity (%)	E-value	Hit length (bp)	Prediction	Score	Prediction	Score	Prediction
4	28,516	26.32	1e−10	513	Temperate	0.3907	Temperate	0.4835	Virulent
12	11,212	27.23	2e−14	606	Temperate	0.3407	Temperate	0.4667	Temperate
52	5,349	29.89	1e−30	798	Temperate	0.0814	Temperate	0.4995	Temperate
88	3,734	24.68	1e−11	828	Temperate	0.2238	Temperate	0.4925	Temperate
173	2,530	26.22	2e−25	1,044	Temperate	0.0691	Virulent	0.5000	Virulent
223	2,233	23.27	1e−12	834	Temperate	0.4770	Virulent	0.5161	Virulent
1257	1,029	29.87	1e−16	462	Virulent	0.7138	Virulent	0.5082	Virulent
1639	921	23.96	2e−11	849	Temperate	0.3558	Temperate	0.4735	Virulent
3299	702	28.18	8e−25	609	Temperate	0.2644	Temperate	0.4744	Temperate
3326	699	30.88	9e−15	405	Temperate	0.3408	Virulent	0.5055	Virulent
6405	549	25.14	1e−13	519	Temperate	0.1150	Virulent	0.5080	Virulent
7704	514	39.86	2e−22	429	Temperate	0.1639	Virulent	0.5110	Temperate
8130	503	36.05	3e−22	441	Temperate	0.0492	Temperate	0.4944	Temperate
9804	470	31.69	2e−16	423	Temperate	0.1605	Temperate	0.4952	Virulent
10819	454	38.61	2e−30	450	Virulent	0.7710	Temperate	0.4951	Virulent
12636	430	34.04	2e−21	417	Virulent	0.7236	Temperate	0.4743	Virulent

“Contig ID" refers to the ID of 16 targeted contigs. “Identity," “E-value," and “Hits length" refer to the alignment results using Blastx.

Furthermere, we found that 16 contigs contain homologous regions of the functional proteins with e−value <1e−10, but they do not have high identity scores to these proteins (identity <50%). These results indicate that the 16 contigs are not close to the viral proteins from the database in genetic relationship. They also show that the diversity of phages in the environmental samples might be much higher than that in the present database, and DeePhage can handle these novel phages. In fact, when we examined the RefSeq viral protein database, we found that a large number of proteins are labelled as “putative” or “hypothetical” and the percentage of such proteins might be much higher than that of bacteria, which further demonstrates the higher diversity of phages.

Not only the several aforementioned contigs but also whole sequences could be considered to examine the full scope of the predictive ability of DeePhage. Using the whole genomes of 77 virulent and 148 temperate phages in Dataset-1, we annotated all the sequences in the virome data of bovine rumen by means of Blastn v2.7.1 [40]. When setting the default parameters (the default e-value is 10), 118,564 contigs could be annotated as virulent or temperate phage genomes by BLAST. Among those contigs, DeePhage distinguishes 56.4% virulent and 48.3% temperate phage contigs with an overall proportion of 51.5%. In comparison, PhagePred and PHACTS seem to prefer virulent contigs (62.3% and 68.2%, respectively) over temperate contigs (39.5% and 28.9%). It is a limitation of those 2 tools that they would miss a large proportion of temperate contigs. Although the proportion of virulent contigs is higher, PhagePred and PHACTS only receive an overall proportion of 48.6% and 44.5%, which is much lower than DeePhage. Estimated on the level of the entire amount of real data, the superiority of DeePhage is certainly considerable.

To sum up, the evaluation of DeePhage using real metavirome data demonstrates that DeePhage makes much better and reliable predictions than PhagePred and PHACTS. As an *ab initio* tool, we can conclude that DeePhage has a good ability to adapt to this diversity and has the potential to analyse newly sequenced phage data.

### An application of a cross-sectional study indicating that phage transformations impact the change of gut microbiota structure

Viruses, especially the phages, contribute importantly to the gut microbiota structure. Particularly, temperate phages could exist free from the genome of their bacterial hosts and then kill them driven by suitable environmental conditions, while virulent phages directly attack their host. Therefore, such phage transformations would change the gut microbiota composition profile and community structure. However, it is hard to analyse this result entirely using the database method because of the limitation of database and marker genes like 16S RNA. As a result, there are no effective computational tools. For example, alignment of phage sequences to the known phage database using the traditional BLAST program could just output some known phages without any new phages. Indeed, the number of unknown phages is huge. Fortunately, DeePhage now could detect phage transformations over the whole genomes of phages from complete virome data. The downstream findings based on DeePhage could give us instructive insights into the function of phages in the gut microbiota.

In this subsection, we then design a new strategy about how to use DeePhage to estimate the transformations of phages in the cross-sectional study. Especially, we analyse the virome data from patients with UC and healthy people as an example to find associations between phages and gut microbiota. For phages in a community, owing to lack of marker genes like 16S RNA to detect their abundance or diversity, it is difficult to determine the association between the transformation of phages and the change of gut microbiota structure. Herein we collected 21 metagenomic samples (randomly selected) of UC patient guts and 21 (randomly selected) metagenomic samples of healthy human guts by Nielsen et al. [41]. In addition, we collected 54 virome samples (viral particles were enriched before sequencing) of UC patient guts (being diagnosed as a specific state) and 23 virome samples of healthy controls by Norman et al. [42]. The accessions (including disease state of virome samples) are provided in Supplementary Tables S2 and S3). We used SPAdes [29] to assemble raw reads of each sample.

For each metagenomic sample, we first used PPR-Meta [11] to identify all the phage-derived contigs. The average percentages of phage contigs in metagenomic data of UC patient and healthy individual guts are similar (23.7% in UC patient and 25.7% in healthy human guts) without significant difference (see Supplementary Fig. 3A, *P*-value = 0.170, the difference in location = 0.021, and 95% confidence interval = (−0.007, 0.045) for 2-sided Wilcoxon rank-sum test). For convenience, in the following text, phage contigs in gut microbiota annotated by PPR-Meta are referred to as computational phages while contigs from virome data are referred to as experimental phages. It is worth noting that experimental phages only included virulent phages and temperate phages in the lytic cycle. However, temperate phages in the lysogenic cycle could not be included because temperate phages in the lysogenic cycle would integrate their genomes into host cells and would not assemble the viral particles. In contrast, computational phages include all kinds of phages.

**Figure 3:**
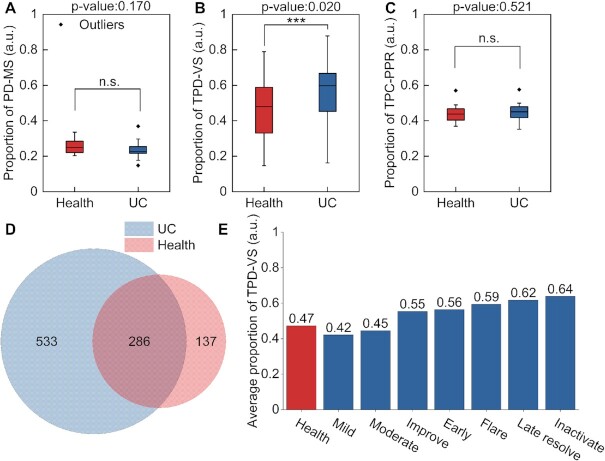
(A) The proportions of phage DNA predicted by PPR-Meta from metagenomic samples of healthy human and UC patient guts are shown using box plots. (B) The proportions of temperate phage DNA predicted by DeePhage from experimental phages (phages from virome samples) of healthy human and UC patient guts are shown using box plots. (C) The proportions of temperate phage DNA predicted by DeePhage from computational phages (phages predicted by PPR-Meta) of healthy human and UC patient guts are shown using box plots. (D) The different species of phages in healthy and UC samples. (E) The average proportion of temperate phages in experimental phages of UC samples. PD-MS: phage DNA from metagenomic samples; TPD-VS: temperate phage DNA from virome samples; TPD-PPR: temperate phage DNA from PPR-Meta; n.s.: no significant difference; ***: significant difference; a.u.: arbitrary unit.

We then used DeePhage to predict the lifestyle of the experimental phages. An average of 56.5% of the contigs are predicted as temperate phages in patients with UC while 47.3% in healthy individuals, with significant difference (see Fig. 3B, *P*-value = 0.020, difference in location = 0.092, and 95% confidence interval = (0.017, 0.170) for 2-sided Wilcoxon rank-sum test). This indicates that the proportion of temperate phages in UC patients' gut is higher than in healthy individuals. However, we still could not infer the detailed transformations from this result because both the decreased richness of virulent phages and increased richness of temperate phages in patients with UC will lead to a higher proportion of temperate phages. More importantly, even if the number of virulent phages and temperate phages is the same in healthy individuals and patients with UC, the proportion of temperate phages in experimental phages could also increase when more temperate phages were undergoing the transformation from the lysogenic cycle to the lytic cycle, in which they would assemble free viral particles. To make the population dynamics clearer, we further used DeePhage to predict the lifestyle of the computational phages. Surprisingly, an average of 44.9% and 44.4% of the contigs are predicted as temperate phages in patients with UC and healthy individuals, respectively, without significant difference (see Fig. 3C, *P*-value = 0.521, the difference in location = –0.01, and 95% confidence interval = (–0.034, 0.017) for 2-sided Wilcoxon rank-sum test), indicating that the proportions of virulent phages and temperate phages in patients with UC and healthy individuals are similar. Considering the results from computational phages and experimental phages together, it seems that the higher proportion of temperate phages among experimental phages of patients with UC might result from the fraction of temperate phages undergoing a transformation from the lysogenic cycle to the lytic cycle. In particular, there is a general tendency that more temperate phages are transforming into the lytic cycle when patients with UC are experiencing more acute disease states. Figure 3E shows the average proportion of temperate phages in experimental phages of patients with UC at different disease states. As we can see, the 2 severe symptom states, “Flare” and “Late resolve”, show higher average proportions, while slight symptoms show lower average proportions, such as “Mild” and “Improve” states.

From these preliminary results, we inferred that the phage populations in patients with UC were undergoing a kind of change that influences the gut microbiota structure, in which some kinds of temperate phages were transforming from prophages to free viral particles. To investigate the transforming temperate phages, we picked out all the temperate contigs annotated by DeePhage from the UC and healthy virome samples. Using all the phage genomes [43] as the database of the BLAST method (e-value ≤ 1e−10), 286 species of phages are existing in both healthy and UC samples, and just 137 species, 98% of which are from the Caudovirales order, only existing in healthy samples (as shown in Fig. 3D). As a comparison, we found different phage contigs coming from 533 species that only exist in UC samples, which probably means that there were more kinds of temperate phages in UC samples than in healthy samples. Those phages could be classified into 10 families: Siphoviridae, Herelleviridae, Podoviridae, Myoviridae, Ackermannviridae, Autographiviridae, Drexlerviridae, Inoviridae, Microviridae, and Sphaerolipoviridae. The first 7 families belong to the Caudovirales order, which accounts for 97% (516 of 533) different species. In addition, a very small proportion (9 different species) is coming from the Microviridae family. The order Caudovirales and family Microviridae are dominant in human gut virome [18]; meanwhile, they are more abundant in patients with UC compared with household members and controls [44]. Especially, Norman et al. observed an increase in the richness of some members of the Caudovirales in patients with UC [42]. This supports our inference to a certain degree. The last several families, which lack researchers’ concerns in the human gut, could roughly be ignored. Because the release of prophages is often associated with the death of bacterial hosts, the activation of the temperate phages may be associated with the change of species composition. We can infer that more kinds of temperate Caudovirales phages switch into a lytic cycle after having the disease and become free viral particles from the bacterial genomes; in consequence, such a switch changes the structure of the microbiota by killing the bacterial host. Consistently, previous research shows that the species compositions of the bacterial community in patients with UC are different from that of healthy individuals [45] and that virulent core phages could be substituted by temperate phages in patients with UC [46]. All those discoveries indicate that maybe it was the temperate Caudovirales phages having a primary impact on human UC disease, which was also verified by us. However, not only could DeePhage detect well-studied phages, such as Caudovirales phages, but it also can trace any known and unknown phages to distinguish their lifestyles. With integrated data, we gain deep access to disease conditions.

To sum up, such a strategy being independent of databases may further provide insights into the specific and integral interactions between phages and bacterial hosts according to phage lifestyles, which could not have been found out before. Researchers can gain more valuable information about the disease process and facilitate the study of human disease.

## Discussion

In this article, we present DeePhage as an effective tool to distinguish virulent phage–derived and temperate phage–derived sequences in metavirome data. Coding a DNA sequence, DeePhage needs no previously extracted features but uses each nucleotide as input. There are some advantages. DeePhage can bypass using the information of some functional genes to make the judgment and directly and rapidly identify each DNA fragment independent of assembly. Such a function is important because many novel phage genomes are difficult to reconstruct and the amount of sequences is large when focused on metagenomic data. Thus, DeePhage can solve the bottleneck of reliably evaluating all phage sequences from metagenomic fragments, while PHACTS, which is based on complete or partial proteome, cannot. CNN models here occupied the core strength of DeePhage for their excellent ability on feature extraction, which is hard to discover by statistics. We have tested that the traditional *k*-mer frequency encoding form was not superior to the one-hot encoding form because the Kmer-4 model produced a terrible prediction as shown in Supplementary Table S1. However, kernels can be seen as position weight matrices to detect motifs, which means that CNN models could still use motifs such as *k*-mer frequencies and perform better. As we can see, DeePhage gradually separates virulent and temperate phage–derived sequences along with deeper neural networks. DeePhage's ability to distinguish 2 kinds of sequences is superior to PhagePred and PHACTS on the assessment of both simulated data and real data. To be specific, DeePhage presents a huge improvement in prediction accuracy (nearly 10% higher and 30% higher on simulated data) and computational efficiency (almost 245 times and 810 times faster). More importantly, DeePhage shed new light on the phage transformations by tracing the variation of a specific type of phage in the human gut. It also demonstrated a possible tendency regarding a larger proportion of temperate phages transforming into the lytic cycle in the gut of patients with UC with a more severe disease state. As we can see, the previous study speculated the possibility that the expansion of the Caudovirales phages is related to the activation of prophages in patients with UC [47]. Fortunately, now we can be more convinced that more temperate Caudovirales phages are entering a lytic cycle. We believe that DeePhage will engender an increasing number of new discoveries, just like the aforementioned problem. Ultimately, DeePhage reduces the reliance on culture-dependent methods and promotes human disease research.

It is also interesting to explore the biological mechanism that helps DeePhage distinguish fragments from these 2 kinds of phage using the sequence signature. In our opinion, this may be because virulent phages and temperate phages face different evolutionary pressures and therefore contain different sequence signatures, such as *k*-mer frequencies as we showed in Supplementary Fig. S1. Genome amelioration often occurs on foreign DNA, such as phage or plasmid, in the host cell, and foreign DNA will change its sequence signatures according to the host chromosome to help it exist stably in the host cell [48]. The similarity of sequence signatures between foreign DNA and bacterial chromosome is often used to predict the bacterial host of the foreign DNA [12,13,48]. Because temperate phages will spend more time in the host cell, they may adjust their sequence signatures toward host chromosomes. Related research also shows that temperate phages do contain more similar sequence signatures to their hosts than virulent phages [22,49]. Therefore, we consider that the difference of sequence signatures played an important role in DeePhage's ability to identify these 2 kinds of phages. To further prove this conjecture, we collected all available bacterial reference genomes (120 bacterial genomes in total) from the RefSeq database [50] (the accession numbers can be seen in Supplementary Table S4) and then used MetaSim to extract artificial contigs between 100 and 1,800 bp. We observed how DeePhage would judge these bacterial sequences. Although DeePhage's training set did not contain any bacterial sequences, DeePhage identifies 74.5%, 77.2%, 71.8%, and 76.9% of the bacterial sequences as temperate phages in Group A, B, C, and D, respectively (the sequence length in each group corresponds to Table 1). We consider that the reason why more than half of the bacterial sequences are identified as temperate phages is that the bacteria contained sequence signatures similar to those of temperate phages. This phenomenon also demonstrates that using the information of sequence signatures may be the working principle of DeePhage. More importantly, we tried to find out some specific sequence features that DeePhage learned. From all the protein sequences of phages in Dataset-1, we picked out a highly trusty set of 3,993 virulent protein sequences and 5,530 temperate protein sequences (prediction score is >0.75 for virulent and <0.25 for temperate by DeePhage). Using the Clusters of Orthologous Genes (COG) database to annotate those proteins, virulent and temperate protein sequences show different distributions in 26 COGs (see Supplementary Fig. S4). Virulent protein sequences show a peak at the “Replication, recombination and repair" category, while temperate protein sequences show a peak at the “Mobilome: prophages, transposons" category. The different distributions may reflect the extracted features of sequences, which are learned by DeePhage and are helpful for this classification task. As we can see in Supplementary Fig. S4, the biggest difference between virulent and temperate protein sequences is located in the “Mobilome: prophages, transposons" category. Temperate phages could exist as prophages and mediate horizontal gene transfer via transduction [51], while such genetic exchanges might be rare when involving virulent phages [52]. Thus, such differences could be comprehended and obviously learned by DeePhage.

DeePhage also has some limitations. Although prokaryotic viruses are dominant in virome samples, a few eukaryotic viruses could also be included. However, DeePhage cannot identify these sequences before distinguishing the lifestyle of each contig. Fortunately, the related tool that helps to distinguish prokaryotic and eukaryotic viruses has been developed recently [53] and we are also considering constructing a preprocessing module for DeePhage to filter out the eukaryotic viruses so that DeePhage can generate more reliable results for the downstream analysis. Furthermore, database biases of different phage species naturally existed in our dataset, for most of the species coming from the Myoviridae, Podoviridae, and Siphoviridae families in the order Caudovirales. It is one of our limitations that the accuracy would be higher for sequences from those families than other families. However, those 3 families are the most abundant among known phages in the human gut [1]; such biases would not seriously affect our performance on those phages. We also believe that this situation will be improved with more accurate labels for phages’ lifestyle.

In conclusion, DeePhage is a novel tool that can quickly and directly judge each fragment as a virulent phage–derived or temperate phage–derived sequence for virome data. Therefore, DeePhage is expected to be a powerful tool for researchers who are interested in the function of phage populations and phage-host interactions.

## Availability of Supporting Source Code and Requirements

Project name: DeePhage

Project home page: http://cqb.pku.edu.cn/ZhuLab/DeePhage or https://github.com/shufangwu/DeePhage

Operating system: The code of DeePhage was written on Linux. We optimized the program in a virtual machine; thus, DeePhage is platform independent.

Programming language: Python, MATLAB

Other requirements: None if running in the virtual machine. If not, Python 3.6.7, TensorFlow 1.4.0, Keras 2.1.3, numpy 1.16.4, h5py 2.9.0, and MATLAB Component Runtime 2018a (for free) are needed. MATLAB is not necessary.

License: GPL-3.0

RRID: SCR_019243

## Data Availability

The artificial contigs, related scripts, and original results are available at http://cqb.pku.edu.cn/ZhuLab/DeePhage/data/ or https://github.com/shufangwu/DeePhage. All the other data are available at corresponding references mentioned in the main text. Snapshots of our code and other data further supporting this work are openly available in the *GigaScience* repository, GigaDB [54].

## Additional Files

Supplementary Fig. S1. The PCA of 4-mer frequency distribution among virulent and temperate phage genomes

Supplementary Fig. S2. The architectures of 6 different models

Supplementary Fig. S3. The ROC curves and AUC scores of DeePhage performance in each set of 5-fold cross-validation

Supplementary Fig. S4. Virulent and temperate proteins show different distributions in 26 COGs

Supplementary Table S1. The Sn, Sp, and Acc of 6 different models

Supplementary Table S2. The accession numbers of 21 metagenomic samples of the healthy human gut and 21 metagenomic samples of UC patients' gut

Supplementary Table S3. The accession numbers of 23 virome samples of the healthy human gut and 54 virome samples of UC patients' gut (with the disease state information)

Supplementary Table S4. The accession numbers of 120 bacterial genomes from RefSeq database

Supplementary Data 1. The detailed information of phage genomes and hosts of phages used in DeePhage from Dataset-1 and Dataset-2.

## Abbreviations

Acc: accuracy: AUC: area under the curve; BLAST: Basic Local Alignment Search Tool; bp: base pairs; CDS: coding sequence; CNN: convolutional neural network; COG: Clusters of Orthologous Genes; CPU: central processing unit; FN: false negative; FP: false positive; NCBI: National Center for Biotechnology Information; PCA: principal component analysis; ROC: receiver operating characteristic; TN: true negative; TP: true positive; Sn: sensitivity; Sp: specificity; t-SNE: t-distributed stochastic neighbour embedding; UC: ulcerative colitis.

## Competing Interests

The authors declare that they have no competing interests.

## Funding

This work was supported by the National Key Research and Development Program of China (2017YFC1200205) and the National Natural Science Foundation of China (32070667, 31671366).

## Authors' Contributions

H.Z. and S.W. proposed and designed the study. J.T. constructed the datasets. S.W. and Z.F. optimized the code. M.L., C.W., and Q.G. contributed to the analysis. C.X. and X.J. helped to test the results. S.W. and H.Z. wrote and revised the manuscript, and all authors proofread and improved the manuscript.

## Supplementary Material

giab056_GIGA-D-20-00378_Original_Submission

giab056_GIGA-D-20-00378_Revision_1

giab056_GIGA-D-20-00378_Revision_2

giab056_Response_to_Reviewer_Comments_Original_Submission

giab056_Response_to_Reviewer_Comments_Revision_1

giab056_Reviewer_1_Report_Original_Submission Satoshi Hiraoka -- 1/25/2021 Reviewed

giab056_Reviewer_1_Report_Revision_1 Satoshi Hiraoka -- 5/21/2021 Reviewed

giab056_Reviewer_2_Report_Original_Submission Sidi Chen -- 2/11/2021 Reviewed

giab056_Reviewer_2_Report_Revision_1 Sidi Chen -- 6/25/2021 Reviewed

giab056_Supplemental_Files
