# Supplementary material for: DeePhage: distinguishing virulent and temperate phage-derived sequences in metavirome data with a deep learning approach
Source: Gigascience. 2021 Sep 8;10(9):giab056. doi: 10.1093/gigascience/giab056 (PMC8427542; doi:10.1093/gigascience/giab056)
Supplement: giab056_GIGA-D-20-00378_Revision_2 [file giab056_giga-d-20-00378_revision_2.pdf]

GigaScience

DeePhage: distinguish virulent and temperate phage-derived sequences in metavirome data with a deep learning approach

--Manuscript Draft--

**Manuscript Number:**	GIGA-D-20-00378R2
**Full Title:**	DeePhage: distinguish virulent and temperate phage-derived sequences in metavirome data with a deep learning approach
**Article Type:**	Research
**Funding Information:**	National Key Research and Development Program of China (2017YFC1200205) — Prof. Huaiqiu Zhu<br>National Natural Science Foundation of China (32070667,31671366) — Prof. Huaiqiu Zhu
**Abstract:**	Background: Prokaryotic viruses referred to as phages can be divided into virulent and temperate phages. Distinguishing virulent and temperate phage-derived sequences in metavirome data is important for their different roles in interactions with bacterial hosts and regulations of microbial communities. However, there is no experimental or computational approach to effectively classify their sequences in culture-independent metavirome. We present a new computational method DeePhage, which can directly and rapidly judge each read or contig as a virulent or temperate phage-derived fragment. Findings: DeePhage utilizes a “one-hot” encoding form to represent DNA sequences in detail. Sequence signatures are detected via a convolutional neural network to obtain valuable local features. The accuracy of DeePhage on five-fold cross validation reaches as high as 89%, nearly 10% and 30% higher than that of two similar tools, PhagePred and PHACTS. On real metavirome, DeePhage correctly predicts the highest proportion of contigs when using BLAST as annotation, without apparent preferences. Besides, DeePhage reduces running time than PhagePred and PHACTS by 245 and 810 times under the same computational configuration. By direct detection of the temperate viral fragments from metagenome and metavirome, we furthermore propose a new strategy to explore phage transformations in the microbial community. The ability to detect such transformations provides us a new insight into the potential treatment for human disease. Conclusions: DeePhage is a novel tool developed to rapidly and efficiently identify two kinds of phage fragments especially for metagenomics analysis. DeePhage is freely available via http://cqb.pku.edu.cn/ZhuLab/DeePhage or https://github.com/shufangwu/DeePhage.
**Corresponding Author:**	Huaiqiu Zhu<br><br>CHINA
**Corresponding Author Secondary Information:**	
**Corresponding Author's Institution:**	
**Corresponding Author's Secondary Institution:**	
**First Author:**	Shufang Wu
**First Author Secondary Information:**	
**Order of Authors:**	Shufang Wu<br>Zhencheng Fang<br>Jie Tan<br>Mo Li<br>Chunhui Wang<br>Qian Guo

Powered by Editorial Manager® and ProduXion Manager® from Aries Systems Corporation

	Congmin Xu
	Xiaoqing Jiang
	Huaiqiu Zhu
**Order of Authors Secondary Information:**	
**Response to Reviewers:**	To Reviewer 1:<br>General Comments: The manuscript was much improved from the last one. The response to the reviewer's concerns has greatly strengthened the manuscript. I have a few additional (unimportant) recommendations as follows.<br>We thank Reviewer 1 for his/her careful reading and efforts to improve our manuscript. It's glad to see that our previous responses were adequately addressed all the points raised by Reviewer 1. The positive comment that "The manuscript was much improved from the last one. The response to the reviewer's concerns has greatly strengthened the manuscript" is really encouraging. The remaining comments raised by Reviewer 1 would help a lot for improving the quality of the manuscript. Below, we itemize our revisions in response to Reviewer 1's points.<br><br>Minor comments:<br>Minor comment 1: Table 2. Hits length -> Hit length (bp)<br>We thank Reviewer 1 for this comment. We have changed "Hits length" to "Hit length (bp)" in Table 2 in the revised manuscript. (Please refer to Line 419, Page 15 in the revised manuscript.)<br><br>Minor comment 2: Figure S1. Check upper/lower cases in labels of x- and y-axes. "First Principal Component" -> "First principal component", and "Second Principal Component" -> "Second principal component"<br>We thank Reviewer 1 for noting the typos in Figure S1. In Figure S1, we have corrected "First Principal Component" to "First principal component" and "Second Principal Component" to "Second principal component". (Please refer to Figure S1 in the revised Additional File 1.)<br><br>Minor comment 3: Additional file 2. Taxonomic columns would be sorted in decreasing order (i.e., Kingdom, Phylum, Class, Order, Family, Genus, Species).<br>We thank Reviewer 1 for reminding us to sort the taxonomic columns in decreasing order. In the revised Additional File 2, we have sorted all the phage and its host taxonomic columns in decreasing order. (Please refer to the revised Additional File 2.)<br><br>To Reviewer 2:<br>General Comments: Overall, the authors have developed a deep learning based tool for classifying phage genomes fragments as a virulent or temperate, with an expanded dataset and several new analyses in this revision. The authors also perform additional analysis.<br>First, there is no point-by-point response to previous round of reviewer's comments, which is essential. From reading of R1 itself it looks like the authors made efforts to address previous comments, however it's not elaborated in response.<br>While the tool may be useful, there are still some reservations regarding the quality of the data both for training and downstream analyses purposes. The reviewer understand that some of the limitations are due to the limitations in the data currently available.<br>We thank Reviewer 2 for the comments to the last round review response. We are pleased to receive the positive judgement as 'Overall, the authors have developed a deep learning based tool for classifying phage genomes fragments as a virulent or temperate, with an expanded dataset and several new analyses in this revision'. Also, we appreciate that Reviewer 2 understood some of our limitations are owing to the data limitations. We believe that such limitations will be improved by the future development of high-quality data. In addition, we have tried our best to present our answers to Reviewer 2's all concerns point-by-point in the last revision, while Reviewer 2 mentioned that 'there is no point-by-point response to previous round of reviewer's comments', and Editor has noticed that there might be some misunderstandings and had emailed Reviewer 2 with our last response separately. Herein we thank both Editor and Reviewer 2 for their understanding our work seriously and responsibly. We then report our responses to Reviewer's further comments as follows.

	Specific comments1: The authors discuss the difference between DeePhage and PHACTs, emphasizing the focus of their tool to be based on metagenomic fragments. They also introduce discussions about and comparisons to the new tool PhagePred. The reviewer acknowledge the value of developing a classifier for phage lifestyle based on metagenomic data, but the main concern was that the dataset (e.g. phage genomes and lifestyle annotations) overlapped exactly with the McNair dataset. To this point, the authors curated another dataset (termed "Dataset-2") that included more phage RefSeq genomes from the NCBI database. The authors write that they "used all Dataset-2 and four fifths of Dataset-1 as the training set, and one fifth of Dataset-1 as the test set" - how was the input dataset partitioned? Were the test set selected randomly? Were they selected by genome or by short sequences? Notably, "Additional File 2" do not have genomes labelled by whether they were used for test or training class. If they used different partitions for cross-validations and the full dataset for final model, this should be stated explicitly. In fact, it would be helpful if the authors provide figures detailing basic statistics (e.g. labels, partitions, lineages, etc.) on their datasets.<br>We are glad to hear that Reviewer 2 admit the value of our work, which is different from PHACTS as we have denoted in the last round review response. However, Reviewer 2 was confused about the usage of new dataset (Dataset-2), we realize that we have not clarified this important point. Therefore, we would like to describe our detailed data processing process point-by-point.<br>(1) “how was the input dataset partitioned?”, “Were the test set selected randomly? Were they selected by genome or by short sequences?”<br>All Dataset-2 and four fifths of Dataset-1 were used as training set and one-fifth of Dataset-1 were used as test set. In detail, the partition of Dataset-1 for training and test is randomly selected by genome. We did a five-fold cross validation as follows. Dataset-1 (225 phage genomes) was randomly separated into five parts. Each part was then used as test set in each cross validation and the rest were used as the training set. Statistically, there are nearly 1820 (180 from four-fifth Dataset-1 and 1640 from all Dataset-2) phage genomes for training and 45 (one-fifth Dataset-1) phage genomes for test in each rotation. Thus, each phage genome in Dataset-1 is used as the test set one time only. Then the MetaSim software was used to simulate 80000 and 20000 short fragments for training and test. To clarify this point, we revised the sentence “Therefore, we used all phages of Dataset-2 and four-fifth phages of Dataset-1 to be the training set, and one-fifth phages of Dataset-1 to be the test set.” to “Therefore, based on genomes, we used all phages of Dataset-2 and four-fifth phages of Dataset-1 to be the training set, and one-fifth phages of Dataset-1 to be the test set.” (Please refer to Line 146-148, Page 5 in the revised manuscript.). More information about the data processing is described in the manuscript as “To test whether DeePhage can distinguish the lifestyle of novel phages or not, for each validation, we divided the training set and the test set based on complete genomes rather than artificial contigs, and then simulated 80,000 training sequences and 20,000 test ones using MetaSim [26].” (Please refer to Line 293-296, Page 11 in the revised manuscript.)<br>(2) “Additional File 2’ do not have genomes labelled by whether they were used for test or training class.”<br>To be clearer and in accordance with the Reviewer’s concerns, we added the usage label (Training usage or Test usage) of each phage genomes in Additional File 2. Usage labels indicate in which cross validation the phage genome was used for training or test set. We added the sentence “Moreover, a usage label (Training usage or Test usage) was used to indicate in which cross validation the phage genome was used for training or test set in Additional File 2.” (Please refer to Line 149-151, Page 6 in the revised manuscript.) Also, we added the column of usage label for each phage genomes in Additional File 2. (Please refer to the revised Additional File 2.)<br>(3) “it would be helpful if the authors provide figures detailing basic statistics (e.g. labels, partitions, lineages, etc.) on their datasets.”<br>Labels and partitions information has been added into Additional File 2 according to items (1) and (2). As for the information of lineages, our previous manuscript has included the lineages information of each phage genomes in Additional File 2. Also, we have sorted all the phage and its host taxonomic columns in decreasing order following the suggestion of Reviewer 1. (Please refer to the revised Additional File 2.)<br><br>Specific comments2: Considering both the significant difference in size and nature of Dataset-1 and 2, and therefore the initial pipeline and the revised one, the authors should compare (at least internally) and clarify which dataset was used for which

Powered by Editorial Manager® and ProduXion Manager® from Aries Systems Corporation

	analyses. Notably: Have the entire pipeline, subsequent analyses (e.g. PCAs), and github implementations described in the main text been updated with combined dataset? How does the performance change between Dataset-1 only and the combined dataset?<br>The PCA analysis did not update, since we still used phage genomes only from Dataset-1. To be clearer, we revised the sentence "…, among virulent phage genomes and temperate phage genomes" to "…, among virulent phage genomes and temperate phage genomes from Dataset-1." (Please refer to Line 168-169, Page 6 in the revised manuscript.)<br>Internally, DeePhage's performance improved on five-fold cross validation set after using the combined dataset, compared with using only Dataset-1. In each length group, the Acc criterion increased 1~2%. It means dataset expanding is useful. For the remain analysis, the performance of DeePhage using the combined dataset slightly altered and have no effects on any conclusion. Thus, we updated all the results using combined dataset in the current manuscript, except for the PCA analysis. Those updated results include analysis on five-fold cross validation, all phage CDS sequences, metavirome from bovine rumen dataset, metagenome/metavirome from healthy/ulcerative-colitis-patients and so on. Those changes were also reported in the response to Reviewer 2's comment #1 in the last round review response.<br><br>Specific comments3: The authors write "We have tested that the traditional k-mer frequency encoding form was not superior to the one-hot encoding form." - can they elaborate on this?<br>As shown in Figure S2 of the previous Additional File 1, we designed a Kmer-4 model, which used k-mer frequencies as an encoding representation, and the architecture of Kmer-4 model was similar to that of DeePhage except to the input layer. Also, we presented that the Kmer-4 model did a terrible prediction as shown in Table S1 of the previous Additional File 1.<br>Thus, to be clearer, we revised the sentence "We have tested that the traditional k-mer frequency encoding form was not superior to the one-hot encoding form." to "We have tested that the traditional k-mer frequency encoding form was not superior to the one-hot encoding form, since Kmer-4 model did a terrible prediction as shown in Additional File 1 (Table S1)." (Please refer to Line 536-537, Page 19 in the revised manuscript.)<br><br>Specific comments4: The authors find differential category distribution according to the COG database for proteins predicted for virulent or temperate phages - the authors should contextualize their findings with what is known in the literature.<br>We thank Reviewer 2 for this suggestion. As we can see in Figure S4, the biggest difference between virulent and temperate protein sequences is located at 'Mobilome: prophages, transposons' category". Temperate phages could exist as prophages and mediate horizontal gene transfer via transduction, while such genetic exchanges might be rare when involving virulent phages. Thus, such the difference could be comprehended and obviously learned by DeePhage. We added the sentence "As we can see in Figure S4, the biggest difference between virulent and temperate protein sequences is located at 'Mobilome: prophages, transposons' category. Temperate phages could exist as prophages and mediate horizontal gene transfer via transduction [51], while such genetic exchanges might be rare when involving virulent phages [52]. Thus, such difference could be comprehended and obviously learned by DeePhage." in the revised manuscript (Please refer to Line 584-588, Page 21 in the revised manuscript.)
**Additional Information:**	
**Question**	**Response**
Are you submitting this manuscript to a special series or article collection?	No
**Experimental design and statistics**<br><br><br>Full details of the experimental design and statistical methods used should be given in the Methods section, as detailed in our	Yes

Minimum Standards Reporting Checklist. Information essential to interpreting the data presented should be made available in the figure legends.<br><br>Have you included all the information requested in your manuscript?	
**Resources**<br><br>A description of all resources used, including antibodies, cell lines, animals and software tools, with enough information to allow them to be uniquely identified, should be included in the Methods section. Authors are strongly encouraged to cite Research Resource Identifiers (RRIDs) for antibodies, model organisms and tools, where possible.<br><br>Have you included the information requested as detailed in our Minimum Standards Reporting Checklist?	Yes
**Availability of data and materials**<br><br>All datasets and code on which the conclusions of the paper rely must be either included in your submission or deposited in publicly available repositories (where available and ethically appropriate), referencing such data using a unique identifier in the references and in the "Availability of Data and Materials" section of your manuscript.<br><br>Have you have met the above requirement as detailed in our Minimum Standards Reporting Checklist?	Yes

Manuscript with DOI Click here to access/download;Manuscript;DeePhage_R2_revised WITH

DeePhage: distinguish virulent and temperate phage-derived sequences in metavirome data with a deep learning approach

Shufang Wu[1,2], Zhencheng Fang[1,2], Jie Tan[1,2], Mo Li[3], Chunhui Wang[3], Qian Guo[1,2,4], Congmin Xu[1,2,4], Xiaoqing Jiang[1,2] and Huaiqiu Zhu[1,2,4,5]*

[1] State Key Laboratory for Turbulence and Complex Systems and Department of Biomedical Engineering, College of Engineering, Peking University, Beijing 100871, China

[2] Center for Quantitative Biology, Peking University, Beijing 100871, China

[3] Peking University-Tsinghua University - National Institute of Biological Sciences (PTN) joint PhD program, School of Life Sciences, Peking University, Beijing 100871, China

[4] Department of Biomedical Engineering, Georgia Institute of Technology and Emory University, Georgia 30332, USA

[5] Institute of Medical Technology, Peking University Health Science Center, Beijing 100191, China.

* To whom correspondence should be addressed. Tel: 8610-6276 7261; Email: hqzhu@pku.edu.cn

ORCID IDs of authors:

Huaiqiu Zhu [0000-0002-6376-218X];

Shufang Wu [0000-0001-7793-2509];

ZhenchenFang [0000-0002-0137-4683];

Jie Tan [0000-0002-5231-2124];

Mo Li [0000-0002-6496-9756];

Chunhui Wang [0000-0001-8292-4896];

Qian Guo [0000-0002-8622-8553];

Congmin Xu [0000-0003-3616-4309];

Xiaoqing Jiang [0000-0001-7475-1630];

Background: Prokaryotic viruses referred to as phages can be divided into virulent and temperate phages. Distinguishing virulent and temperate phage-derived sequences in metavirome data is important for their different roles in interactions with bacterial hosts and regulations of microbial communities. However, there is no experimental or computational approach to effectively classify their sequences in culture-independent metavirome. We present a new computational method DeePhage,

which can directly and rapidly judge each read or contig as a virulent or temperate phage-derived fragment. **Findings:** DeePhage utilizes a “one-hot” encoding form to represent DNA sequences in detail. Sequence signatures are detected via a convolutional neural network to obtain valuable local features. The accuracy of DeePhage on five-fold cross validation reaches as high as 89%, nearly 10% and 30% higher than that of two similar tools, PhagePred and PHACTS. On real metavirome, DeePhage correctly predicts the highest proportion of contigs when using BLAST as annotation, without apparent preferences. Besides, DeePhage reduces running time than PhagePred and PHACTS by 245 and 810 times under the same computational configuration. By direct detection of the temperate viral fragments from metagenome and metavirome, we furthermore propose a new strategy to explore phage transformations in the microbial community. The ability to detect such transformations provides us a new insight into the potential treatment for human disease. **Conclusions:** DeePhage is a novel tool developed to rapidly and efficiently identify two kinds of phage fragments especially for metagenomics analysis. DeePhage is freely available via http://cqb.pku.edu.cn/ZhuLab/DeePhage or https://github.com/shufangwu/DeePhage.

INTRODUCTION

In a microbial community, phages are the major component of the viral genetic materials. It is estimated that the number of phages is on average ten times higher than that of bacteria [1]. They may destroy bacteria, meanwhile in some situations benefit populations of bacteria, and thus crucially impact the microbial community [2]. With the development of the high-throughput sequencing technology, a large number of novel phages are discovered from metagenomes and viromes, in which viral particles are first enriched before sequencing [3,4]. However, the analysis of these phage sequences is a great challenge since the reference genomes of phages are very limited in view of the fact that most of the phages cannot be cultured independently. The complete phage genomes in current databases are much less than that of bacteria, therefore a large number of sequences from virome data cannot find regions with homology to the known phages [3]. In addition, unlike bacteria, phages lack the universal marker genes such as 16S rRNA [5], so that many species identification strategies designed for bacterial analysis are not applicable to phages. Moreover, for mobile elements such as phages, the sequence assembly of them is often poorer than that of bacteria, usually because the mobile elements carry repetitive regions like insertion sequences and share sequences that occurred among different

genomes [6]. As a result, the large number of short fragments in metagenomic data also increases the difficulty of the analysis.

To overcome these difficulties, several computational tools focusing on two major tasks have been developed to analyze the phage sequences from metagenome or virome. One of the tasks is to identify phage fragments in metagenomic data, such as the tools VirSorter [7], VirFinder [8], MARVEL [9], virMine [10], and PPR-Meta [11]. Especially, PPR-Meta is a tool with high performances developed by us and demonstrates much better accuracy than the related tools. Another task is to assign the host for a given phage contig, such as the tools WIsH [12], VirHostMatcher [13], and Hostphinder [14]. However, these tools cannot answer the question about how the newly discovered phages interact with their hosts. According to the interaction mode, which is also referred to as the phage lifestyle, phages can be divided into the virulent phages and the temperate phages [15]. When a virulent phage infects its host, it will produce many progenies as soon as the phage's DNA is injected into the host cell and then causes the death of the host through bacterial lysis [15]. In contrast, temperate phages can undergo the lysogenic cycle and lytic cycle. In the lysogenic cycle, a temperate phage will integrate its genome into the host chromosome, which is also referred to as a kind of prophage, and then copies its genome together with the host chromosome [1]. While induced by appropriate conditions, especially the nutritional conditions and the number of co-infecting phages, temperate phages will go into the lytic cycle, followed by releasing the viral particle and killing the host through bacterial lysis [16]. Such different processes have a significant influence on the microbiota especially in the human gut, which could be highly correlated with human diseases or the treatment of human disease. Although some kinds of hotspots, such as phage therapies that make use of the virulent phages in the context of therapeutic use [17], have been investigated, limited by current bioinformatics tools, people still knew little about these different lifecycles for their prevalence in the human gut [18]. Therefore, it is important to distinguish virulent and temperate phages for further understanding of phage-host interaction.

Although the classification strategy of this issue for virome data is still a challenge, there are several noteworthy works that help to characterize the virulent and temperate phages. Even phages lacking marker genes, those studies show that they may have some functional genes, which are high-frequency genes and can tell us whether a given phage is virulent or temperate in a relatively credible way. For example, Emerson et al. found there were some functional genes for temperate phages, such as integrase and excisionase [19]; Schmidt et al. found that the leucine substitution in DNA *pol*A gene had

a strong connection with temperate phages [20]. Notably, McNair et al. design a computational tool called PHACTS to identify whether a phage with a complete or partial proteome is virulent or temperate [15]. This tool employs all the sequence information of proteins from a phage genome and uses the random forest as a classifier to make the judgment. Researchers further found that the existence of some kind of genes helped PHACTS present good results. For example, virulent phages usually have genes related to phage lysis, nucleotide metabolism, or structural proteins, while temperate phages usually contain genes related to toxins, excision, integration, lysogeny, or regulation of expression [15]. Unfortunately, such kind of strategies may not apply to metagenomic data. To date, it is still a difficult task to reconstruct complete genomes of all organisms in the metagenomic data. Therefore, only a few DNA fragments may contain those functional genes that can help to make the judgment. According to the report of PHACTS, this tool can achieve accuracy over 95% if at least 25 proteins are provided from a phage genome. However, if fewer proteins are obtained, the accuracy of PHACTS reduces obviously. When only five proteins are obtained from a phage, PHACTS only achieves an accuracy of about 65%; if only two proteins are obtained from a phage, PHACTS appears to produce random results with an accuracy below 55%. Considering that most of the DNA sequences in metagenomic data are short fragments that only contain a few genes or even incomplete genes, it is essential to develop a tool, which does not depend on information from sufficient proteins with functional genes level, while to make judgment directly for each short DNA fragment in metagenomic data. Recently, a new tool PhagePred was developed to identify phages' lifestyle in metagenomic sequences [21]. Based on the Markov model, PhagePred uses k-mer frequencies as the sequence feature to test the dissimilarity measures between new contig and two kinds of phages. Then it is used to determine the new contig's lifestyle. PhagePred was tested on various contig lengths (500, 1000, 3000, 5000, and 10,000bp). However, as a global statistic, such k-mer methods will generate much noise with a short metagenomic sequence. When PhagePred involves in sequences from 500bp to 10,000bp, for a short sequence, the k-mer frequencies may be different from long sequences, and the variance may be higher than long sequences. Thus, the k-mer based method may not be applicable for all ranges of contig length for PhagePred. Another concern is that k-mer frequency will lose detailed sequence information when encoding sequence to k-mer feature vectors. Considering all shortcomings mentioned above, it is better to develop a new method that is more suitable for short phage sequences.

In this paper, we present a two-class classifier DeePhage to identify whether a DNA fragment is derived from a virulent phage or a temperate phage. Using the information of every nucleotide without manually feature extracted, DeePhage encodes sequences in “one-hot” form. Such representations are suitable for the Convolutional Neural Network (CNN) model to detect helpful motifs for classification, which are common used on biological sequence identification. Together with other kinds of neural network layers, DeePhage learns different features between virulent and temperate sequences and then outputs a score indicating the possibility to be a certain kind of phage sequence. Tested on the same data, DeePhage can significantly outperform the related method PhagePred and PHACTS on computational efficiency by using only 1/245 and 1/810 computation time that PhagePred and PHACTS uses. Simulation tests on five-fold validation show that DeePhage precedes in accuracy metrics by approximately 10% and 30% compared with PhagePred and PHACTS. DeePhage’s evaluations on real metavirome data of bovine rumen are better than PhagePred and PHACTS with much more accurate results, which use annotations of the BLAST method as a relatively accurate reference. Meanwhile, we present a new strategy to conveniently detect the phage transformation by tracing specific types of phage contigs, which can explore the influence of phages that contribute to microbial communities even to human diseases. DeePhage can be used to analyse the virome data and metagenomic data directly. While handling the metagenomic data, users need to firstly identify the phage sequences using related software, such as PPR-Meta [11] as we mentioned above, and then use DeePhage to further annotate the phage sequences.

MATERIAL AND METHODS

Data construction

Considering that there is no real virome data with the reliable lifestyle annotation for each sequence as a benchmark, we constructed artificial contigs extracted from well-annotated complete phage genomes as the benchmark to train and test the algorithm. We download 227 complete phage genomes with lifestyle annotations from McNair dataset, including 79 virulent phages and 148 temperate phages[15]. Among these phages, we removed two virulent phages from the dataset: mycobacteriophage D29 (accession: NC_001900) and lactococcus lactis bacteriophage ul36 (accession: NC_004066), because the lifestyle of these two phages may be ambiguous. Although these two phages are annotated as virulent phages, researchers found that they both contained functional integrases, indicating that they

can integrate their genomes into host chromosomes like temperate phages [15]. Besides, D29 is very similar to the temperate phage L5 [22], while ul36 has 46.6% homology with the temperate phage Tuc2009 [23]. Therefore, 77 virulent phages and 148 temperate ones are used in the current study, named Dataset-1. What's more, following the phage lifestyle dataset constructing strategy of Song [21], we recruited more than 1500 phage RefSeq genomes from the NCBI database [24]. Their lifestyle annotations are labelled using a bioinformatic method [25]. Excluding the overlapped phages in Dataset-1, we included 1211 virulent and 429 temperate phage genomes (Dataset-2). Dataset-1 is manually curated with credible lifestyle annotations, while Dataset-2 is not. Therefore, based on genomes, we used all phages of Dataset-2 and four-fifth phages of Dataset-1 to be the training set, and one-fifth phages of Dataset-1 to be the test set. Detailed information of each phage genome and their host information from Dataset-1 and Dataset-2 were shown in Additional File 2. Moreover, a usage label (Training usage or Test usage) was used to indicate in which cross validation the phage genome was used for training or test set in Additional File 2. For convenience, herein the virulent phages are referred to as the positive sample and the temperate phages as the negative sample.

We further used MetaSim (v0.9.1) [26] to extract artificial contigs from the complete phage genomes. Considering that the length of contigs in real metagenomes may cover a wide range, we divided the artificial contigs into four groups according to their length: the length range in Group A is 100-400 bp; Group B is 400-800 bp; Group C is 800-1200 bp while Group D is 1200-1800 bp. Those four groups may cover the length of raw reads and the average length of assembled contigs from the next-generation sequencing technology. We would evaluate the performance of DeePhage on different groups respectively.

We also used real virome data to estimate the reliability of DeePhage qualitatively. We downloaded virome data of bodily fluid in the bovine rumen [27] from MG-RAST [28]. They were downloaded as raw reads (accessions: mgm4534202.3 and mgm4534203.3). We used SPAdes (v3.13.0) [29] to assemble the raw reads and obtained 118918 contigs with the N50 of 291 bp.

Mathematical model of phage sequences

To evaluate the feasibility of the sequence signature used for classifying virulent and temperate phages, we first analysed the distribution of k-mer frequencies, which have been widely used to distinguish genomes from different species, among virulent phage genomes and temperate phage genomes from

Dataset-1. Using 4-mer frequencies with anosim (Analysis of Similarities) test shows that there is a significant difference between those two groups (R = 0.080, P = 0.001), which means that they have different sequence signatures to characterize the two categories of phage genomes. To be visualized, we did Principal Component Analysis (PCA) [30] based on 4-mer frequencies features (see Figure S1 in Additional File 1).

Although k-mer frequencies have shown their ability to classify virulent and temperate phage genomes, using such frequencies to characterize short DNA fragments will usually be disturbed with the noise [12]. Also, as global statistics that may miss some local information, k-mer frequencies are difficult to detail characterize mobile elements that contain mosaic structure [31]. To describe the local sequence information in detail, we consider the one-hot encoding form, which can represent every base continuously and entirely. For each sequence, we used the "one-hot" encoding form to represent each base in a sequence. Specifically, bases A, C, G and T were represented by [0,0,0,1], [0,0,1,0], [0,1,0,0], and [1,0,0,0]. In particular, the one-hot encoding form could be regarded as a special 1-mer-frequency detector.

Algorithm structure of DeePhage

Deep learning algorithms are recognized as an extremely effective method in many fields including in the biology field. Comparing with the Recurrent Neural Network (RNN), the CNN models are faster to train and more efficient in sequential spatial correlations [32]. Specifically, CNN is a universal network for extracting local patterns in terms of biology, which in the current context can be used as a motif detector of DNA sequences. In DeePhage, we present a deep learning algorithm with CNN models to handle the input sequences represented by the one-hot encoding form. The network contains eight layers: a 1D convolutional (Conv1D) layer, a 1D maximum pooling (Maxpooling) layer, a 1D global average pooling (Globalpooling) layer, two batch normalization (BN1 and BN2) layer, a dropout (Dropout) layer, and two dense (Dense1 and Dense2) layers.

Conv1D layer takes a sequence encoded by an $\mathrm{L} \times 4$ matrix X (L is the length of the sequences, equals 400, 800, 1200, and 1800) as the input and generates total F feature maps as output by corresponding F convolutional kernels. Those kernels can be used to detect vital motifs. Using ReLU (Rectified Linear Unit) [33] as the activation function, the Conv1D layer output an $\mathrm{L} \times \mathrm{F}$ matrix $Y^C$and computes for the fth feature map at the lth location like this:

$$Y_{\mathrm{l,f}}^{C} = \ \mathrm{ReLU}(\sum_{m=0}^{\mathrm{M\text{-}1}}\sum_{n=0}^{3} W_{m,n}^{\mathrm{f}} X_{\mathrm{l}+m,n} + b_{\mathrm{f}}^{\mathrm{C}}),$$

$$\text{for } \mathrm{l} = 0,1,2,\ldots,\mathrm{L}-1, \mathrm{f} = 0,1,2,\ldots,\mathrm{F}-1.$$

The W^{f} and $b_{\mathrm{f}}^{\mathrm{C}}$ are an M×4 weight matrix and a bias of the fth kernel. The mentioned ReLU function is defined as [33]:

$$\mathrm{ReLU}(\mathrm{x}) = \begin{cases} \mathrm{x} & \text{if } \mathrm{x} \geq 0 \\ 0 & \text{if } \mathrm{x} < 0 \end{cases}.$$

As a traditional nonlinear function, the ReLU function is easier to train and achieves better performance, which can rectify the shortcomings of sigmoid functions. Those kernels scan a sequence one after another to extract the valuable features for the classification and the ReLU function achieves a nonlinear transformation.

Such a combination is followed by the Maxpooling layer to downsample the input representation by taking the maximum value over an input channel with a pooling size S1 and a stride size S2. The window is shifted along with each channel independently and can generate F new channels with the size of $\mathrm{L}^{'}$ ($\mathrm{L}^{'} = \mathrm{L}/S2$). The Maxpooling layer outputs an $\mathrm{L}^{'} \times \ \mathrm{F}$ feature matrix Y^{M} and one of the pooling operation for a specific channel at the lth location defines like this:

$$Y_{\mathrm{l,f}}^{\mathrm{M}} = \max(Y_{\mathrm{l\times S2,f}}^{\mathrm{C}}, Y_{\mathrm{l\times S2+1,f}}^{\mathrm{C}}, Y_{\mathrm{l\times S2+2,f}}^{\mathrm{C}}, \ldots, Y_{\mathrm{l\times S2+S1\text{-}1,f}}^{\mathrm{C}}) \ ,$$

$$\text{for } \mathrm{l} = 0,1,2,\ldots,\mathrm{L}^{'}-1\ ,\ \ \mathrm{f} = 0,1,2,\ldots,\mathrm{F}-1.$$

Its main function is to reduce the dimensions of each input channel using final summarised features, which can also adapt to location variations of valuable features.

Features from the Maxpooling layer are passed to the BN1 layer to scale the inputs. At each batch, it usually transforms inputs to have a mean close to 0 and a standard deviation close to 1, which can avoid the vanishing gradient problem and accelerate the convergence rate of the model. Thus, the output feature matrix Y^{B1} of the BN1 layer is also an $\mathrm{L}' \times \ \mathrm{F}$ matrix as Y^{M} but being scaled.

The next is a Dropout layer, which randomly drops a certain proportion (denoted as P) of input elements by setting them to zero during training [32]. The output $\boldsymbol{Y}^{\mathrm{Dp}}$ is formulated as:

$$\boldsymbol{Y}^{\mathrm{Dp}} = \ \mathbf{K} \odot \boldsymbol{Y}^{\mathrm{B1}}, \text{ where } \mathbf{K} \sim B(1, \mathrm{P}).$$

The drop mask $\mathbf{K}$ denotes a Bernoulli distribution with *n* equals 1 and *p* equals P. It could effectively reduce overfitting especially in our small dataset [34].

After a dropout layer, the Globalpooling layer takes the $\boldsymbol{Y}^{\mathrm{Dp}}$ as input and reduce features from the same channel into one dimension by using the average value of those features, which can integrate global spatial information. More formally:

$$y_{\mathrm{f}}^{\mathrm{G}} = \frac{1}{\mathrm{L}'} \sum_{l=0}^{\mathrm{L}'-1} Y_{l,\mathrm{f}}^{\mathrm{Dp}}, \text{for } \mathrm{f} = 0,1,2,\ldots,\mathrm{F}-1,$$

where y_{f}^{G} is the average value of features from the fth input channel. Considering all the F channels from the previous layer, the output of the Globalpooling layer $\boldsymbol{y}^{\mathrm{G}}$ is an F dimension vector.

Subsequently, a Dense1 layer using ReLU function as activation function outputs R units. It has an R×F weight matrix W^{D1} and an R-dimensional bias vector b^{D1}. Each output units is processed by:

$$y_{\mathrm{r}}^{\mathrm{D1}} = \mathrm{ReLu}\Big(\sum_{\mathrm{f}=0}^{\mathrm{F}-1} W_{\mathrm{r,f}}^{\mathrm{D1}}\, y_{\mathrm{f}}^{\mathrm{G}} + b_{\mathrm{r}}^{\mathrm{D1}}\Big), \text{for } \mathrm{r} = 0,1,2,\ldots,\mathrm{R}-1.$$

Dense1 layer can compile the features from different input channels together and finally generate an R-dimensional vector $\boldsymbol{y}^{\mathrm{D1}}$, while a Conv1D layer just extracts features into different feature maps.

The vector $\boldsymbol{y}^{\mathrm{D1}}$is then sent into a BN2 layer to generate a new feature vector $\boldsymbol{y}^{\mathrm{B2}}$ that having a mean close to 0 and a standard deviation close to 1, which has the same effect as the BN1 layer.

Using a sigmoid function as an activation function, the final layer is the Dense2 layer and output only one score between zero and one representing the probability of prediction. Using an R-dimensional weight vector W^{D2} and a bias scalar b^{D2}, the output score is given by:

$$y^{\mathrm{D2}} = \mathrm{sigmoid}\Big(\sum_{r=0}^{\mathrm{R}-1} W_r^{\mathrm{D2}}\, y_r^{\mathrm{B2}} + b^{\mathrm{D2}}\Big).$$

The sigmoid function is defined as:

$$\mathrm{sigmoid(x)} = \frac{1}{1+\mathrm{e}^{-\mathrm{x}}}$$

In default, the sequence with a score higher than 0.5 would be regarded as a positive sample (a virulent phage) and the sequence with a score lower than 0.5 would be regarded as a negative sample (a temperate phage). When training, we used the Adam optimizer [35] (learning rate = 0.0001), binary cross-entropy as the loss function, and 32 as the batch size to train the neural network and update network weights. Altogether, we found that setting the size F to 64, M to 6, S1 to 3, S2 to 3, P to 0.3, and R to 64 made the best performance. The structure of DeePhage is shown in the upper part of Figure 1.

It is worthy to know more about the importance of the encoding method for sequences and each specific layer in our model, so we tested six different models (including DeePhage) by using k-mer frequencies as an encoding representation or removing a certain layer. The six model architectures (DeePhage, Kmer-4, No-Maxpooling, No-Dropout, No-Globalpooling, and No-BN) are shown in Additional File 1 (Figure S2) and their performances are shown in Additional File 1 (Table S1). It can be seen that the Kmer-4 model did a terrible prediction. As mentioned above, when we used 4-mer frequencies to characterize each phage at the level of genome sequences, it could slightly distinguish two kinds of phages. It was proved that k-mer frequencies had no enough power to represent short sequences and are fit for capturing the global signature of long sequences rather than the local signature of short sequences. Thus, we used convolutional kernels to detect sequence motifs, where each column of kernels represents the probabilities of having A/C/G/T at one position (position weight matrices (PWM)) [36]. Those kernels can involve k-mer frequencies but in a more detailed and local way. As for those models removing a certain layer, the performance dropped compared with DeePhage. Especially, the prediction accuracy reduced nearly 14% and 5% when using a model without a Globalpooling layer and BN layers (No-Globalpooling and No-BN model). Other models decreased slightly. We can see the architecture and the one-hot encoding representation of DeePhage are better than others.

Although deep neural networks are considered as black-box models, we hope to have insights into the learning process for features. We chose five layers (One-hot input, Conv1D, BN1, Globalpooling, and BN2) to observe their learned features. Because it is hard to gain an intuitive display about high-dimension features, we used t-Distributed Stochastic Neighbor Embedding (t-SNE) [37], which is a machine learning algorithm for dimensionality reduction, for the visualization of high-dimensional data in a 2D projected space. After the training process, we firstly used PCA to reduce features of the five layers mentioned above into a 10-dimensional space and then used t-SNE to reduce them into a two-dimensional space using the sequences from Group D. The visualizations of the five layers are shown in the lower part of Figure 1. It could be seen that the effects of classification are better when focusing on the deeper layers. In detail, two types of phages were firstly mixture together and then separated gradually, which demonstrated the learning process of DeePhage. Furthermore, it should be emphasized that the visualizations by dimensionality reduction cannot reflect the complete power of DeePhage.

Considering the other length of sequences beyond our four-trained groups, we design some strategies. For those sequences longer than 1800 bp, DeePhage will split the sequence into several 1800-bp-long subsequences without overlapping, usually except the last subsequence. DeePhage will then use the neural network in the corresponding group to predict each subsequence, and calculate the weighted average score according to the score and length of each subsequence. Because training the neural network using long sequences is very time-consuming, we do not train additional neural networks for longer sequences. For those sequences shorter than 100 bp, DeePhage uses the neural network in Group A to predict.

RESULTS

Identification performance of DeePhage, PhagePred and PHACTS

We first used the five-fold cross validation to evaluate the performance of DeePhage. To test whether DeePhage can distinguish the lifestyle of novel phages or not, for each validation, we divided the training set and the test set based on complete genomes rather than artificial contigs, and then simulated 80,000 training sequences and 20,000 test ones using MetaSim [26]. The performance evaluation criteria here are defined as: $Sn=TP/(TP+FN)$; $Sp=TN/(TN+FP)$; and $Acc=(TP+TN)/(TP+TN+FN+FP)$. Among these criteria, *Sn* and *Sp* are used to evaluate the accuracy of virulent phages and temperate phages respectively, while *Acc* is used to evaluate the overall performance. As shown in Table 1, DeePhage demonstrates overall reliable and stable performance with *Acc* from 76% to 89%. Compared with PhagePred, the *Acc* criteria of PhagePred are 11~14% lower than DeePhage, as shown in Table 1. We used d_2^* dissimilarity measures, k-mer length of 9 and Markov order of 2 as prediction parameters. Although *Sn* are close to DeePhage, *Sp* are more than 18% lower than DeePhage. It is probably owing to some mentioned-above limitations when applied k-mer based method to short sequences. Thus, the performance of DeePhage is superior to PhagePred. Compared with PHACTS, the *Acc* of PHACTS are only around 50%, which is also lower than DeePhage. For PHACTS, sequences are inputted in form of amino acid sequences and sequences without coding region are determined as wrong predictions. Such results indicate that the input of functional genes with several proteins is not required for our DeePhage. Therefore, our DeePhage method shows an evident advantage compared with the tool PHACTS. Since DeePhage can identify each DNA fragment as the virulent phage-derived sequence or temperate phage-derived one directly and independently, it would be a more acceptable

tool to analyse phages in metagenomic data. In this case, the complete or near-complete genomes for phages are hard to be reconstructed from the data, especially for those with low abundance or in a low coverage sequencing condition. Clearly, our DeePhage has the advantage of being applicable to processing the data by current short-read sequencing technologies and performs better when the short reads could be assembled into longer contigs. More details about the performances of the ROC curves and AUC scores of DeePhage in each rotation of the five-fold cross validation are shown in Additional File 1 (Figure S3).

In general, sequences with scores near 0.5 are not as reliable as those sequences with a score near 0 or 1. Therefore, DeePhage is designed with an adjustable cutoff to filter out these uncertain predictions. Users can specify a cutoff using a parameter. In this way, a sequence with a score between (0.5-cutoff/2, 0.5+cutoff/2) will be labelled as "uncertain". In general, with a higher cutoff, the percentage of uncertain predictions will be higher while the remaining predictions will be more reliable. As a recommendation, we suggest that 0.50 is a suitable value of cutoff. It means a sequence with a score between (0.25, 0.75) would better be ignored. When test on the cross validation set, it can receive a high average AUC score and remain enough sequences. If hoping for a more reliable prediction, users could assign the parameter 't' to 0.50 as told in the manual of DeePhage. While using 0.50 as cutoff, the main results have no changes for analyzing real dataset. Thus, we do not use a cutoff in the following analysis.

Table 1. Results of five-fold cross validation for DeePhage, PhagePred and PHACTS. The validation of each group was performed independently. Each result consists of the mean and standard deviation.

Tools	Criteria	Group A (100-400 bp)	Group B (400-800 bp)	Group C (800-1200 bp)	Group D (1200-1800 bp)
DeePhage	*Sn(%)*	77.3±4.2	82.2±3.4	86.2±3.2	87.5±3.3
	Sp(%)	74.6±6.9	84.4±8.2	86.3±10.2	89.5±8.5
	Acc(%)	76.2±1.9	83.7±2.5	86.6±3.6	88.9±2.9
PhagePred	*Sn(%)*	75.8±1.4	80.8±1.9	84.2±2.1	87.4±2.0
	Sp(%)	56.6±5.0	60.3±6.2	62.8±7.2	64.7±7.8
	Acc(%)	65.7±2.4	70.1±2.9	72.9±3.5	75.5±3.6
PHACTS	*Sn(%)*	73.7±0.7	64.7±1.0	65.8±1.6	69.1±1.7
	Sp(%)	26.3±1.5	36.9±1.2	39.1±0.3	42.3±2.4
	Acc(%)	48.6±1.7	49.9±1.2	51.5±0.8	54.8±1.5

Comparison with PhagePred and PHACTS for protein sequence identification

It should be noted that DeePhage and PHACTS are designed for different tasks, PHACTS is designed for complete genomes while DeePhage is designed for metagenomic fragments. Therefore, the

requirements of the input data for them are actually different. PHACTS requires users to input all proteins (amino acid sequences) within one phage genome, so proteins from different phages should not be put into the same file. In contrast, the DeePhage's requirement is only to input all DNA fragments (nucleic acid sequences), no matter whether they contain coding regions and whether they are from the same phage, and DeePhage may directly judge each fragment independently. Although it was difficult to compare three tools based on the same condition, we tried to test the performance of PHACTS only in DNA short fragments with coding region. Since PHACTS requires a collection of protein sequences as input, we firstly annotated the protein sequences of 100,000 DNA sequences of the test set in each length group using FragGeneScan (v1.31) [38] and proteins from the same sequence (sequences without coding regions were ignored) are input into the program PHACTS (v0.3). As for comparison, DeePhage and PhagePred are also used to predict these DNA sequences with coding regions. The total accuracy (the number of correct predictions divided by the total number of sequences having the coding regions in each length group) of DeePhage, PhagePred and PHACTS in each length group are shown in Figure 2. For short fragments covering data sets of Group A to D, PHACTS demonstrates the accuracies of *Acc* around 50%, which are nearly the results of random predictions. In Group D, PhagePred demonstrates the accuracy of *Acc* around 76% and still 13% lower than that of DeePhage. In contrast, DeePhage can satisfactorily classify the sequences with the accuracies of *Acc* about 76%~89%.

In addition, we have evaluated the performances of DeePhage, PhagePred and PHACTS on the coding sequences (CDSs) from all 225 phage genomes. Since PHACTS could only process protein sequences, we extracted all CDSs from the genomes according to the GenBank annotation and each CDS was independently inputted to PHACTS (in the form of amino acid sequences), PhagePred (in the form of nucleic acid sequences) and DeePhage (in the form of nucleic acid sequences). We found that PHACTS can only achieve the *Acc* of 54.3%, which is also near to random judgment results, while DeePhage achieves the *Acc* of 82.7%, nearly 30% higher than that of PHACTS. PhagePred achieves the *Acc* of 68.4%, which is also lower than DeePhage. Considering that the number of CDSs in each metagenomic fragment is very limited, PHACTS has actually a very limited ability to analyse metagenomic data especially when the complete genomes could not be reconstructed using these fragments. Overall, as a state-of-the-art tool designed for phage lifestyle classification from

metagenomic data, DeePhage, a de novo tool using the deep learning algorithm, presents efficient prediction.

Also, DeePhage can handle large scale high-throughput data within an acceptable running time. In order to test, we recorded the runtime of DeePhage, PhagrPred and PHACTS to predict 100 DNA sequences (converted to protein sequences for PHACTS) ranging from 100-1800 bp. DeePhage spends nearly 10 seconds which is 245 times faster than PhagePred and 810 times faster than PHACTS (using nearly 41 minutes and 135 minutes), when tested on a virtual machine with the following configuration: CPU: Intel Core i7 4790; and Memory: 8G, DDR3. As for PHACTS, every sequence needs to be aligned and every prediction needs to be replicated ten times, while DeePhage could directly predict every sequence without any alignments. Therefore, DeePhage is much faster than PHACTS.

Evaluation of DeePhage, PhagePred and PHACTS using real metavirome data

Although it was difficult to make exact evaluations using real data, some functional genes could help us to make an approximately effective assessment of our model. In this subsection, we used DeePhage to predict all the sequences in a metavirome data of bovine rumen [27] with 118918 contigs assembled by SPAdes (v3.13.0) [29]. As a result, 53.6% (63691/118918) of the contigs are predicted as virulent phage-derived contigs and 46.4% (55227/118918) as temperate phage-derived contigs. For assessment of the DeePhage's prediction, we then collected the RefSeq viral protein database [39] as a reference. Since the viral proteins labelled of 'excision', 'integration', or 'lysogeny' are more likely to exist in temperate phages [15], we used those proteins to build an MTPD (mini temperate phage-derived) set containing 107 protein sequences. We then searched all 118918 contigs against the MTPD data using Blastx v2.7.1[40] and obtained 16 targeted contigs having homologous regions (e-value $\leq$ 1e−10, hits length $\geq$ 400). These hits present an extremely small proportion (16/118918), which confirms that there are a huge number of data having no reliable homologous regions of known databases. When it comes to DeePhage, 13 of 16 targeted contigs can be identified as temperate phage-derived contigs, while only 6 contigs and 10 contigs can be classified as temperate phage-like contigs by PhagePred and by PHACTS. It shows DeePhage performs better than PhagePred and PHACTS and has rather the potential to analyze newly sequenced phage data. However, the prediction scores being nearly 0.5 shows PHACTS actually made randomly inferring, while DeePhage having a majority of reliable scores

and making better predictions. The information of 16 targeted contigs and predicted results by DeePhage, PhagePred and PHACTS are listed in Table 2.

Further, we found that 16 contigs contain homologous regions of the functional proteins with the *e*-value lower than 1e-10, but they do not have high identity scores to these proteins (identity<50%). These results indicate that the 16 contigs are not close to the viral proteins from the database in the genetic relationship. These also show that the diversity of phages in the environmental samples might be much higher than that in the current database, and DeePhage can handle these novel phages. In fact, when we looked over the RefSeq viral protein database, we found that a large number of proteins are labelled as "putative" or "hypothetical" and the percentage of such proteins might be much higher than that of bacteria, which further demonstrates the higher diversity of phages.

Not only the several above-mentioned contigs but also the whole sequences could be taken into consideration in a full scope of the predictive ability of DeePhage. Using the whole genomes of 77 virulent and 148 temperate phages in Dataset-1, we annotated all the sequences in the virome data of bovine rumen by Blastn v2.7.1[40]. When setting the default parameters (the default e-value is 10), 118564 contigs could be annotated as virulent or temperate phage genomes by BLAST. Among those contigs, DeePhage distinguishes 56.4% virulent and 48.3% temperate phage contigs with an overall proportion of 51.5%. In comparison, PhagePred and PHACTS make an apparent preference for virulent contigs (62.3% and 68.2%) and temperate contigs (39.5% and 28.9%). It is a limitation for those two tools that they would miss a large part of temperate contigs. Although the proportion of virulent contigs is higher, PhagePred and PHACTS only receive an overall proportion of 48.6% and 44.5%, which is much lower than DeePhage. Estimated on the level of entire real data, the superiority of DeePhage is certainly considerable.

Table 2. Information of 16 targeted contigs and predicted results by DeePhage, PhagePred and PHACTS. **'**Contig ID' refers to the ID of 16 targeted contigs. 'Identity', 'E-value', and 'Hits length' refer to the alignment results using Blastx.

Contig ID	Contig length (bp)	Identity (%)	E-value	Hit length(bp)	DeePhage prediction	score	PHACTS prediction	score	PhagePred prediction
4	28516	26.32	1e-10	513	temperate	0.3907	temperate	0.4835	virulent
12	11212	27.23	2e-14	606	temperate	0.3407	temperate	0.4667	temperate
52	5349	29.89	1e-30	798	temperate	0.0814	temperate	0.4995	temperate
88	3734	24.68	1e-11	828	temperate	0.2238	temperate	0.4925	temperate

173	2530	26.22	2e-25	1044	temperate	0.0691	virulent	0.5000	virulent
223	2233	23.27	1e-12	834	temperate	0.4770	virulent	0.5161	virulent
1257	1029	29.87	1e-16	462	virulent	0.7138	virulent	0.5082	virulent
1639	921	23.96	2e-11	849	temperate	0.3558	temperate	0.4735	virulent
3299	702	28.18	8e-25	609	temperate	0.2644	temperate	0.4744	temperate
3326	699	30.88	9e-15	405	temperate	0.3408	virulent	0.5055	virulent
6405	549	25.14	1e-13	519	temperate	0.1150	virulent	0.5080	virulent
7704	514	39.86	2e-22	429	temperate	0.1639	virulent	0.5110	temperate
8130	503	36.05	3e-22	441	temperate	0.0492	temperate	0.4944	temperate
9804	470	31.69	2e-16	423	temperate	0.1605	temperate	0.4952	virulent
10819	454	38.61	2e-30	450	virulent	0.7710	temperate	0.4951	virulent
12636	430	34.04	2e-21	417	virulent	0.7236	temperate	0.4743	virulent

To sum up, the evaluation of DeePhage using real metavirome data demonstrates DeePhage makes much better and reliable predictions than PhagePred and PHACTS. As an ab initial tool, it may be concluded that DeePhage has a good ability to adapt to this diversity and has the potential to analyse newly sequenced phage data.

An application of a cross-sectional study indicating that phage transformations impacting the change of gut microbiota structure

Viruses especially the phages contribute importantly to the gut microbiota structure. Particularly, temperate phages could be free from the genome of their bacterial hosts and then kill them driven by a suitable environment condition, while virulent phages directly attack their host. Therefore, such phage transformations would change the gut microbiota composition profile and community structure. However, it is hard to analyse this result entirely using the databased method because of the limitation of database and marker genes like 16S RNA. As a result, there are no effectively computational tools. For example, alignment phage sequences to the known phage database using the traditional Blast program could just output some known phages without any new phages. Indeed, the number of unknown phages are extraordinarily huge. Fortunately, DeePhage now could detect phage transformations over the whole genomes of phages from the complete virome data. The downstream findings based on DeePhage could give us instructive insights into the function of phages in the gut microbiota.

In this subsection, we then design a new strategy about how to use DeePhage to estimate the transformations of phages in the cross-sectional study. Specially, we analyse the virome data from

ulcerative colitis (UC) patients and healthy people as an example to find out associations between phages and gut microbiota. For phages in a community, owing to lack of marker genes like 16S RNA to detect their abundance or diversity, it is difficult to determine the association between the transformation of phages and the change of gut microbiota structure. Herein we collected 21 metagenomic samples (randomly selected) of UC patient guts and 21 (randomly selected) metagenomic samples of healthy human guts by Nielsen et al. [41]. In addition, we collected 54 virome samples (viral particles were enriched before sequencing) of UC patient guts (being diagnosed as a specific state) and 23 virome samples of healthy control by Norman et al. [42]. The accessions (including disease state of virome samples) are provided in Additional File 1 (Table S2 and S3). We used SPAdes [29] to assemble raw reads of each sample.

For each metagenomic sample, we first used PPR-Meta [11] to identify all the phage-derived contigs. The average percentage of phage contigs in metagenomic data of UC patient and healthy individual guts are similar (23.7% in UC patient and 25.7% in healthy human guts) without significant difference (see Figure 3A, p-value=0.170, the difference in location=0.021 and 95% confidence interval = (–0.007, 0.045) for two-sided Wilcoxon Rank-Sum test). For convenience, in the following text, phage contigs in gut microbiota annotated by PPR-Meta are referred to as computational phages while contigs from virome data are referred to as experimental phages. It is worth noting that experimental phages only included virulent phages and temperate phages in the lytic cycle. However, temperate phages in the lysogenic cycle could not be included, because temperate phages in the lysogenic cycle would integrate their genomes into host cells and would not assemble the viral particles. In contrast, computational phages include all kinds of phages.

We then used DeePhage to predict the lifestyle of the experimental phages. An average of 56.5% of the contigs are predicted as temperate phages in UC patients while 47.3% in healthy individuals with significant difference (see Figure 3B, p-value=0.020, difference in location=0.092 and 95% confidence interval = (0.017, 0.170) for two-sided Wilcoxon Rank-Sum test). This indicates that the proportion of temperate phages in UC patients' gut is higher than in healthy individuals. However, we still could not infer the detailed transformations from this result, because both the decreased richness of virulent phages and increased richness of temperate phages in UC patients will lead to a higher proportion of temperate phages. More importantly, even if the number of virulent phages and temperate phages is the same in healthy individuals and UC patients, the proportion of temperate phages in experimental

phages could also increase when more temperate phages were undergoing the transformation from the lysogenic cycle to the lytic cycle, in which they would assemble free viral particles. To make the population dynamics clearer, we further used DeePhage to predict the lifestyle of the computational phages. Surprisingly, an average of 44.9% and 44.4% of the contigs are predicted as temperate phages in UC patients and healthy individuals without significant difference (see Figure 3C, *p*-value=0.521, the difference in location=–0.01 and 95% confidence interval = (–0.034, 0.017) for two-sided Wilcoxon Rank-Sum test), indicating that the proportion of virulent phages and temperate phages in UC patients and healthy individuals are approximate. Considering the results from computational phages and experimental phages together, it seems that the higher proportion of temperate phages in experimental phages of UC patients might result from the part of temperate phages undergoing a transformation from the lysogenic cycle to the lytic cycle. In particular, there is a general tendency that more temperate phages are transforming into the lytic cycle when UC patients are suffering from more acute disease states. Figure 3E shows the average proportion of temperate phages in experimental phages of UC patients at different disease states. As we can see, the two serve symptom, “Flare” and “Late resolve” states, show higher average proportions, while slight symptom shows lower average proportion, such as “Mild” and “Improve” states.

From these preliminary results, we inferred that the phage populations in UC patients were undergoing a kind of change that influence the gut microbiota structure, in which some kinds of temperate phages were transforming from prophages to free viral particles. To investigate the transforming temperate phages, we picked out all the temperate contigs annotated by DeePhage from the UC and healthy virome samples. Using all the phage genomes [43] as the database of the BLAST method (e-value ≤ 1e−10), 286 species of phages are existing in both Healthy and UC samples, and just 137 species, 98% of which are from the *Caudovirales* order, only existing in Healthy samples (as shown in Figure 3D). As a comparison, we found out different phage contigs coming from 533 species that only existing in UC samples, which probably means that there were more kinds of temperate phages in UC samples than in Healthy samples. Those phages could be classified into ten families: *Siphoviridae*, *Herelleviridae*, *Podoviridae*, *Myoviridae*, *Ackermannviridae*, *Autographiviridae*, *Drexlerviridae*, *Inoviridae*, *Microviridae*, *Sphaerolipoviridae*. The first seven families belong to the *Caudovirales* order, which accounts for nearly 97% (516/533) different species. Besides, a very small part (nine different species) is coming from *Microviridae* family. *Caudovirales* order and *Microviridae*

family are dominated in human gut virome [18], meanwhile, they are more abundant in UC patients compared with household members and controls [44]. Especially, Norman et al. observed an increase in the richness of some members of the *Caudovirales* in UC patients [42]. Those supported our inference to a certain degree. The last several families lacking researchers' concerns in the human gut could roughly be ignored. Since the release of prophages is often associated with the death of bacterial hosts, the activation of the temperate phages may be associated with the change of species composition. We can infer that more kinds of temperate *Caudovirales* phages turn into a lytic cycle after having the disease and become free viral particles from the bacterial genomes, in consequence, such switch change the struct of microbiota by killing the bacterial host. Consistently, previous research shows that the species compositions of the bacteria community in UC patients are different from that of healthy individuals [45] and the virulent core phages could be substituted by temperate phages in UC patients [46]. All those discoveries indicated that maybe it was the temperate *Caudovirales* phages having a primary impact on human UC disease, which was also verified by us. However, DeePhage could not only detect well-studied phages, such as *Caudovirales* phages, but it also can trace any known and unknown phages to distinguish their lifestyles. With integrated data, we have access to disease conditions deeply.

To sum up, such a strategy being independent of databases may further provide insights into the specific and integral interactions between phages and bacterial hosts according to phage lifestyles, which could not have been found out before. Researchers can gain more valuable information about the disease process and facilitate the study of human disease.

DISCUSSION

In this paper, we present DeePhage as an effective tool to distinguish virulent phage-derived and temperate phage-derived sequences in metavirome data. Coding a DNA sequence, DeePhage needs no previously extracted features but use each nucleotide as input. There are some advantages. DeePhage can bypass using the information of some functional genes to make the judgment and directly and rapidly identify each DNA fragment being independent of assembling. Such a function is important because many novel phage genomes are difficult to reconstruct and the amount of sequences is large when focused on metagenomic data. Thus, DeePhage can solve the bottleneck to make a reliable evaluation on all phage sequences from metagenomic fragments, while PHACTS based on

complete or partial proteome cannot. CNN models here occupied the core strength of DeePhage for their excellent ability on feature extraction, which is hard to discover by statistics. We have tested that the traditional k-mer frequency encoding form was not superior to the one-hot encoding form, since Kmer-4 model did a terrible prediction as shown in Additional File 1 (Table S1). However, kernels can be seen as position weight matrices to detect motifs, which means CNN models could still employ motifs such as k-mer frequencies and make better performance. As we can see, DeePhage gradually separates virulent and temperate phage-derived sequences along with deeper neural networks. DeePhage's ability to distinguish two kinds of sequences is superior to PhagePred and PHACTS on the assessment of simulated data and real data. To be specific, DeePhage presents a huge improvement in prediction accuracy (nearly 10% higher and 30% higher on simulated data) and computational efficiency (almost 254 times and 810 times faster). More importantly, DeePhage shed new light on the phage transformations by tracing the variation of a specific type of phage in the human gut. It also demonstrated a possible tendency about more temperate phages transforming into the lytic cycle in the gut of UC patients with a more serve disease state. As we can see, the previous study speculated the possibility that the expansion of the *Caudovirales* phages is related to the activation of prophages in UC patients [47]. Fortunately, now we can be more convinced that more temperate *Caudovirales* phages are turning into a lytic cycle. We believe that there will be an increasing number of new discoveries, just like the problem mentioned before, on account of DeePhage. Afterward, DeePhage ultimately reduces the dependency on culture-dependent methods and promotes human disease research.

It is also interesting to explore the biological mechanism that helps DeePhage distinguish fragments from these two kinds of phage using the sequence signature. In our opinion, this may because virulent phages and temperate phages face different evolutionary pressures and therefore contain different sequence signatures, such as k-mer frequencies as we showed in Figure S1. Genome amelioration often occurs on foreign DNA, such as phage or plasmid, in the host cell and foreign DNA will change its sequence signatures according to the host chromosome to help it exist stably in the host cell [48]. The similarity of sequence signatures between foreign DNA and bacterial chromosome is often used to predict the bacterial host of the foreign DNA [12,13,48]. Since temperate phages will spend more time in the host cell, they may adjust their sequence signatures toward host chromosomes. Related researches also show that temperate phages do contain more similar sequence signatures to

their hosts than virulent phages [22,49]. Therefore, we consider that the difference of sequence signatures played an important role for DeePhage to identify these two kinds of phages. To further prove this conjecture, we collected all available bacterial reference genomes (totally 120 bacterial genomes) from the RefSeq database [50] (the accession numbers can be seen in Additional File1, Table S4) and then used MetaSim to extract artificial contigs between 100 to 1800 bp. We observed how DeePhage would judge these bacterial sequences. Although the training set of DeePhage did not contain any bacterial sequences, DeePhage identifies 74.5%, 77.2%, 71.8%, and 76.9% of the bacterial sequences as temperate phages in Group A, B, C, and D, respectively (the sequence length in each group was corresponding to Table 1). We consider that the reason why more than half of the bacterial sequences are identified as temperate phages is that bacteria contained similar sequence signatures with temperate phages. This phenomenon also demonstrates that using the information of sequence signatures may be the working principle of DeePhage. More importantly, we tried to find out some specific sequence features that DeePhage learned. From all the protein sequences of phages in Dataset-1, we picked out highly trusty 3993 virulent protein sequences and 5530 temperate protein sequences (prediction score is bigger than 0.75 for virulent and smaller than 0.25 for temperate by DeePhage). Using the COG database to annotate those proteins, virulent and temperate protein sequences show different distributions in 26 COGs (see Figure S4 in Additional File1). Virulent protein sequences show a pick at 'Replication, recombination and repair' category, while temperate protein sequences show a pick at 'Mobilome: prophages, transposons' category. The different distributions may reflect the extracted features of sequences, which is learned by DeePhage and is helpful for this classification task. As we can see in Figure S4, the biggest difference between virulent and temperate protein sequences is located at 'Mobilome: prophages, transposons' category. Temperate phages could exist as prophages and mediate horizontal gene transfer via transduction [51], while such genetic exchanges might be rare when involving virulent phages [52]. Thus, such difference could be comprehended and obviously learned by DeePhage.

DeePhage also has some limitations. Although prokaryotic viruses are dominant in virome samples, a few eukaryotic viruses could also be included. However, DeePhage cannot identify these sequences before distinguishing the lifestyle of each contig. Fortunately, the related tool that helps to distinguish prokaryotic and eukaryotic viruses has been developed recently [53] and we are also considering constructing a preprocessing module for DeePhage to filter out the eukaryotic viruses so that DeePhage

can generate more reliable results for the downstream analysis. Furthermore, the database biases of different phage species naturally existed in our dataset, for most of the species coming from the *Myoviridae*, *Podoviridae* and *Siphoviridae* families in *Caudovirales* order. It is one of our limitations that the accuracy would be higher for sequences from those families than other families. However, those three families are the most abundant phage families within known phages in the human gut [1]; such biases would not seriously impact our performance on those phages. We also believe that this situation will be indeed improved with more accurate labels for phages' lifestyle.

In conclusion, DeePhage is a novel tool that can directly judge each fragment as a virulent phage-derived or temperate phage-derived sequence for virome data in a fast way. Therefore, it is expected that DeePhage will be a powerful tool for researchers who are interested in the function of phage populations and phage-host interactions.

Availability of supporting source code and requirements

Project name: DeePhage.

Project home page: http://cqb.pku.edu.cn/ZhuLab/DeePhage or https://github.com/shufangwu/DeePhage.

Operating system: The code of DeePhage was written on Linux. We optimized the program in a virtual machine; thus, DeePhage is platform independent.

Programming language: python, matlab

Other requirements: no other requirements are needed if running in the virtual machine. If not, Python 3.6.7, TensorFlow 1.4.0, Keras 2.1.3, numpy 1.16.4, h5py 2.9.0 and MATLAB Component Runtime 2018a (for free) are needed. MATLAB is not necessary.

License: GPL-3.0.

RRID: SCR_019243

Availability of supporting data and materials

The artificial contigs, related scripts, and original results are available at http://cqb.pku.edu.cn/ZhuLab/DeePhage/data/ or https://github.com/shufangwu/DeePhage. All the other data are available at corresponding references mentioned in the main text. Snapshots of our code

and other data further supporting this work are openly available in the GigaScience repository, GigaDB [54].

Additional files

Additional file 1: Figure S1. The PCA of 4-mer frequencies distribution among virulent and temperate phage genomes; Figure S2. The architectures of six different models; Figure S3. The ROC curves and AUC scores of DeePhage performances in each set of five-fold cross validation; Figure S4. Virulent and temperate proteins show different distributions in 26 COGs; Table S1. The *Sn*, *Sp*, and *Acc* of six different models; Table S2. The accession numbers of 21 metagenomic samples of the healthy human gut and 21 metagenomic samples of UC patients' gut; Table S3. The accession numbers of 23 virome samples of the healthy human gut and 54 virome samples of UC patients' gut (with the disease state information); Table S4. The accession numbers of 120 bacterial genomes from RefSeq database.

Additional file 2: The detailed information of phage genomes and hosts of phage used in DeePhage from Dataset-1 and Dataset-2.

Authors' contributions

H.Q.Z. and S.F.W. proposed and designed the study. J.T. constructed the datasets. S.F.W. and Z.C.F. optimized the code. M.L., C.H.W., and Q.G. contributed to the analysis. C.M.X and X.Q.J helped to test the results. S.F.W. and H.Q.Z. wrote and revised the manuscript, and all authors proofread and improved the manuscript.

ACKNOWLEDGEMENT

We thank Dr. Li Qu, Dr. Luotong Wang, Man Zhou and Chuan He of Peking University for their helpful discussions. Part of the analysis was performed on the High Performance Computing Platform of the Center for Life Science of Peking University.

FUNDING

This work was supported by the National Key Research and Development Program of China (2017YFC1200205) and the National Natural Science Foundation of China (32070667, 31671366).

CONFLICT OF INTEREST

The authors declare that they have no competing interests.

TABLE AND FIGURES LEGENDS

Figure 1. Structure of deep learning neural network and visualization of five layers by reducing dimensions. DeePhage uses the CNN model as the classifier. The neural network (in the upper part) takes the sequence in the "one-hot" coding form as input and output a score between zero and one. In general, the sequence with a score higher than 0.5 can be referred to as the virulent phage-derived fragment and the sequence with a score lower than 0.5 can be referred to as the temperate phage-derived fragment. The visualization demonstrated the learning process of DeePhage. The performance would be better when we observing a deeper layer (in the lower part).

Table 1. Results of five-fold cross validation for DeePhage, PhagePred and PHACTS. The validation of each group was performed independently. Each result consists of the mean and standard deviation.

Figure 2. Comparison results of DeePhage, PhagePred and PHACTS in each length group.

Table 2. Information of 16 targeted contigs and predicted results by DeePhage, PhagePred and PHACTS. 'Contig ID' refers to the ID of 16 targeted contigs. 'Identity', 'E-value', and 'Hits length' refer to the alignment results using Blastx.

Figure 3. (A) The proportions of phage DNA predicted by PPR-Meta from metagenomic samples of healthy human and UC patient guts are shown using the box plots; (B) The proportions of temperate phage DNA predicted by DeePhage from experimental phages (phages from virome samples) of healthy human and UC patient guts are shown using the box plots; (C) The proportions of temperate phage DNA predicted by DeePhage from computational phages (phages predicted by PPR-Meta) of healthy human and UC patient guts are shown using the box plots; (D) The different species of phages in Healthy and UC samples; (E) The average proportion of temperate phages in experimental phages of UC samples. Note: PD-MS, phage DNA from metagenomic samples; TPD-VS, temperate phage DNA from virome samples; TPD-PPR, temperate phage DNA from PPR-Meta; n.s., no significant difference.

REFERENCES

1. Mirzaei, M.K. and Maurice, C.F. Menage a trois in the human gut: interactions between host, bacteria and phages. Nat Rev Microbiol. 2017;**15**(7):397-408.
2. Wommack, K.E. and Colwell, R.R. Virioplankton: Viruses in aquatic ecosystems. Microbiol. Mol. Biol. Rev. 2000;**64**(1):69-114.
3. Hayes, S., Mahony, J., Nauta, A. and van Sinderen, D. Metagenomic approaches to assess bacteriophages in various environmental niches. Viruses 2017;**9**(6):127.
4. Paez-Espino, D., Eloe-Fadrosh, E.A., Pavlopoulos, G.A., Thomas, A.D., Huntemann, M., Mikhailova, N., Rubin, E., Ivanova, N.N. and Kyrpides, N.C. Uncovering earth's virome. Nature 2016;**536**(7617):425-430.
5. Mokili, J.L., Rohwer, F. and Dutilh, B.E. Metagenomics and future perspectives in virus discovery. Curr Opin Virol. 2012;**2**(1):63-77.
6. Rozov, R., Kav, A.B., Bogumil, D., Shterzer, N., Halperin, E., Mizrahi, I. and Shamir, R. Recycler: an algorithm for detecting plasmids from de novo assembly graphs. Bioinformatics 2017;**33**(4):475-482.
7. Roux, S., Enault, F., Hurwitz, B.L. and Sullivan, M.B. VirSorter: mining viral signal from microbial genomic data. Peerj 2015;**3**:e985.
8. Ren, J., Ahlgren, N.A., Lu, Y.Y., Fuhrman, J.A. and Sun, F.Z. VirFinder: a novel k-mer based tool for identifying viral sequences from assembled metagenomic data. Microbiome 2017;**5**(1):69.
9. Amgarten, D., Braga, L.P.P., da Silva, A.M. and Setubal, J.C. MARVEL, a tool for prediction of bacteriophage sequences in metagenomic bins. Front Genet. 2018;**9**:304.
10. Garretto, A., Hatzopoulos, T. and Putonti, C. virMine: automated detection of viral sequences from complex metagenomic samples. Peerj 2019;**7**:e6695.
11. Fang, Z.C., Tan, J., Wu, S.F., Li, M., Xu, C.M., Xie, Z.J. and Zhu, H.Q. PPR-Meta: a tool for identifying phages and plasmids from metagenomic fragments using deep learning. Gigascience 2019;**8**(6):giz066.
12. Galiez, C., Siebert, M., Enault, F., Vincent, J. and Soding, J. WIsH: who is the host? Predicting prokaryotic hosts from metagenomic phage contigs. Bioinformatics 2017;**33**(19):3113-3114.
13. Ahlgren, N.A., Ren, J., Lu, Y.Y., Fuhrman, J.A. and Sun, F.Z. Alignment-free d(2)(*) oligonucleotide frequency dissimilarity measure improves prediction of hosts from metagenomically-derived viral sequences. Nucleic Acids Res. 2017;**45**(1):39-53.
14. Villarroel, J., Kleinheinz, K.A., Jurtz, V.I., Zschach, H., Lund, O., Nielsen, M. and Larsen, M.V. HostPhinder: a phage host prediction tool. Viruses 2016;**8**(5):116.
15. McNair, K., Bailey, B.A. and Edwards, R.A. PHACTS, a computational approach to classifying the lifestyle of phages. Bioinformatics 2012;**28**(5):614-618.
16. Erez, Z., Steinberger-Levy, I., Shamir, M., Doron, S., Stokar-Avihail, A., Peleg, Y., Melamed, S., Leavitt, A., Savidor, A., Albeck, S. *et al.* Communication between viruses guides lysis-lysogeny decisions. Nature 2017;**541**(7638):488-493.
17. Brives, C. and Pourraz, J. Phage therapy as a potential solution in the fight against AMR: obstacles and possible futures. Palgrave Commun. 2020;**6**:100.

18. Sutton, T.D.S. and Hill, C. Gut Bacteriophage: current understanding and challenges. Front Endocrinol. 2019;**10**:784.
19. Emerson, J.B., Thomas, B.C., Andrade, K., Allen, E.E., Heidelberg, K.B. and Banfield, J.F. Dynamic viral populations in hypersaline systems as revealed by metagenomic assembly. Appl Environ Microbiol. 2012;**78**(17):6309-6320.
20. Schmidt, H.F., Sakowski, E.G., Williamson, S.J., Polson, S.W. and Wommack, K.E. Shotgun metagenomics indicates novel family A DNA polymerases predominate within marine virioplankton. ISME J. 2014;**8**(1):103-114.
21. Song, K. Classifying the lifestyle of metagenomically-derived phages sequences using alignment-free methods. Front Microbiol. 2020;**11**.
22. Deschavanne, P., Dubow, M.S. and Regeard, C. The use of genomic signature distance between bacteriophages and their hosts displays evolutionary relationships and phage growth cycle determination. Virol J. 2010;**7**:163.
23. Labrie, S. and Moineau, S. Complete genomic sequence of bacteriophage u136: Demonstration of phage heterogeneity within the P335 quasi-species of lactococcal phages. Virology 2002;**296**(2):308-320.
24. The NCBI database. https://www.ncbi.nlm.nih.gov/refseq/. Accessed 1 March 2021
25. Mavrich, T.N. and Hatfull, G.F. Bacteriophage evolution differs by host, lifestyle and genome. Nat Microbiol 2017;**2**:17112.
26. Richter, D.C., Ott, F., Auch, A.F., Schmid, R. and Huson, D.H. MetaSim-a sequencing simulator for genomics and metagenomics. PLoS One 2008;**3**(10):e3373.
27. Ross, E.M., Petrovski, S., Moate, P.J. and Hayes, B.J. Metagenomics of rumen bacteriophage from thirteen lactating dairy cattle. BMC Microbiol. 2013;**13**:242.
28. Meyer, F., Paarmann, D., D'Souza, M., Olson, R., Glass, E.M., Kubal, M., Paczian, T., Rodriguez, A., Stevens, R., Wilke, A. *et al.* The metagenomics RAST server - a public resource for the automatic phylogenetic and functional analysis of metagenomes. Bmc Bioinformatics 2008;**9**:386.
29. Bankevich, A., Nurk, S., Antipov, D., Gurevich, A.A., Dvorkin, M., Kulikov, A.S., Lesin, V.M., Nikolenko, S.I., Pham, S., Prjibelski, A.D. *et al.* SPAdes: a new genome assembly algorithm and its applications to single-cell sequencing. J Comput Biol. 2012;**19**(5):455-477.
30. Wold, S., Esbensen, K. and Geladi, P. Principal component analysis. Chemometr Intell Lab Syst 1987;**2**(1-3):37-52.
31. Ford, M.E., Sarkis, G.J., Belanger, A.E., Hendrix, R.W. and Hatfull, G.F. Genome structure of mycobacteriophage D29: Implications for phage evolution. J Mol Biol. 1998;**279**(1):143-164.
32. Zheng, D.D., Pang, G.S., Liu, B., Chen, L.H. and Yang, J. Learning transferable deep convolutional neural networks for the classification of bacterial virulence factors. Bioinformatics 2020;**36**(12):3693-3702.
33. Agarap, A.F. Deep Learning using Rectified Linear Units (ReLU). arXiv. 2018. https://arxiv.org/abs/1803.08375
34. Srivastava, N., Hinton, G., Krizhevsky, A., Sutskever, I. and Salakhutdinov, R. Dropout: a simple way to prevent neural networks from overfitting. J. Mach. Learn. Res. 2014;**15**(1):1929-1958.

35. Kingma, D. and Ba, J. Adam: a method for stochastic optimization. arXiv. 2014. https://arxiv.org/abs/1412.6980v8
36. Ren, J., Song, K., Deng, C., Ahlgren, N.A., Fuhrman, J.A., Li, Y., Xie, X.H., Poplin, R. and Sun, F.Z. Identifying viruses from metagenomic data using deep learning. Quant Biol 2020;**8**(1):64-77.
37. van der Maaten, L. and Hinton, G. Visualizing data using t-SNE. J. Mach. Learn. Res. 2008;**9**:2579-2605.
38. Rho, M.N., Tang, H.X. and Ye, Y.Z. FragGeneScan: predicting genes in short and error-prone reads. Nucleic Acids Res. 2010;**38**(20):e191.
39. The NCBI database. ftp://ftp.ncbi.nih.gov/refseq/release/viral/. Accessed 6 June 2018
40. Johnson, M., Zaretskaya, I., Raytselis, Y., Merezhuk, Y., McGinnis, S. and Madden, T.L. NCBIBLAST: a better web interface. Nucleic Acids Res. 2008;**36**:W5-W9.
41. Nielsen, H.B., Almeida, M., Juncker, A.S., Rasmussen, S., Li, J.H., Sunagawa, S., Plichta, D.R., Gautier, L., Pedersen, A.G., Le Chatelier, E. *et al.* Identification and assembly of genomes and genetic elements in complex metagenomic samples without using reference genomes. Nat Biotechnol. 2014;**32**(8):822-828.
42. Norman, J.M., Handley, S.A., Baldridge, M.T., Droit, L., Liu, C.Y., Keller, B.C., Kambal, A., Monaco, C.L., Zhao, G., Fleshner, P. *et al.* Disease-specific alterations in the enteric virome in inflammatory bowel disease. Cell 2015;**160**(3):447-460.
43. The NCBI database. ftp://ftp.ncbi.nlm.nih.gov/genomes/GENOME_REPORTS/. Accessed 23 November 2020
44. Scarpellini, E., Ianiro, G., Attili, F., Bassanelli, C., De Santis, A. and Gasbarrini, A. The human gut microbiota and virome: Potential therapeutic implications. Dig Liver Dis. 2015;**47**(12):1007-1012.
45. Qin, J.J., Li, R.Q., Raes, J., Arumugam, M., Burgdorf, K.S., Manichanh, C., Nielsen, T., Pons, N., Levenez, F., Yamada, T. *et al.* A human gut microbial gene catalogue established by metagenomic sequencing. Nature 2010;**464**(7285):59-65.
46. Clooney, A.G., Sutton, T.D.S., Shkoporov, A.N., Holohan, R.K., Daly, K.M., O'Regan, O., Ryan, F.J., Draper, L.A., Plevy, S.E., Ross, R.P. *et al.* Whole-virome analysis sheds light on viral dark matter in inflammatory bowel disease. Cell Host Microbe 2019;**26**(6):764-778.e765.
47. Mukhopadhy, I., Segal, J.P., Carding, S.R., Hart, A.L. and Hold, G.L. The gut virome: the 'missing link' between gut bacteria and host immunity? 2019. doi: 10.1177/1756284819836620
48. Suzuki, H., Yano, H., Brown, C.J. and Top, E.M. Predicting plasmid promiscuity based on genomic signature. J Bacteriol. 2010;**192**(22):6045-6055.
49. Ahmed, S., Saito, A., Suzuki, M., Nemoto, N. and Nishigaki, K. Host-parasite relations of bacteria and phages can be unveiled by oligostickiness, a measure of relaxed sequence similarity. Bioinformatics 2009;**25**(5):563-570.
50. Pruitt, K.D., Tatusova, T. and Maglott, D.R. NCBI Reference Sequence (RefSeq): a curated non-redundant sequence database of genomes, transcripts and proteins. Nucleic Acids Res. 2005;**33**:D501-D504.
51. Monteiro, R., Pires, D.P., Costa, A.R. and Azeredo, J. Phage Therapy: Going Temperate? Trends Microbiol. 2019;**27**(4):368-378.

52. Moura de Sousa, J.A., Pfeifer, E., Touchon, M. and Rocha, E.P.C. Causes and Consequences of Bacteriophage Diversification via Genetic Exchanges across Lifestyles and Bacterial Taxa. Mol Biol Evol. 2021;38(6):2497-2512.

53. Galan, W., Bak, M. and Jakubowska, M. Host taxon predictor - a tool for predicting taxon of the host of a newly discovered virus. Sci Rep. 2019;9(1):3436.

54. Wu S; Fang Z; Tan J; Li M; Wang C; Guo Q; Xu C; Jiang X; Zhu H: Supporting data for "DeePhage: distinguish virulent and temperate phage-derived sequences in metavirome data with a deep learning approach" GigaScience Database. 2021. http://dx.doi.org/10.5524/100918

Figure_1_revised

Click here to access/download;Figure;Figure_1_revised.png

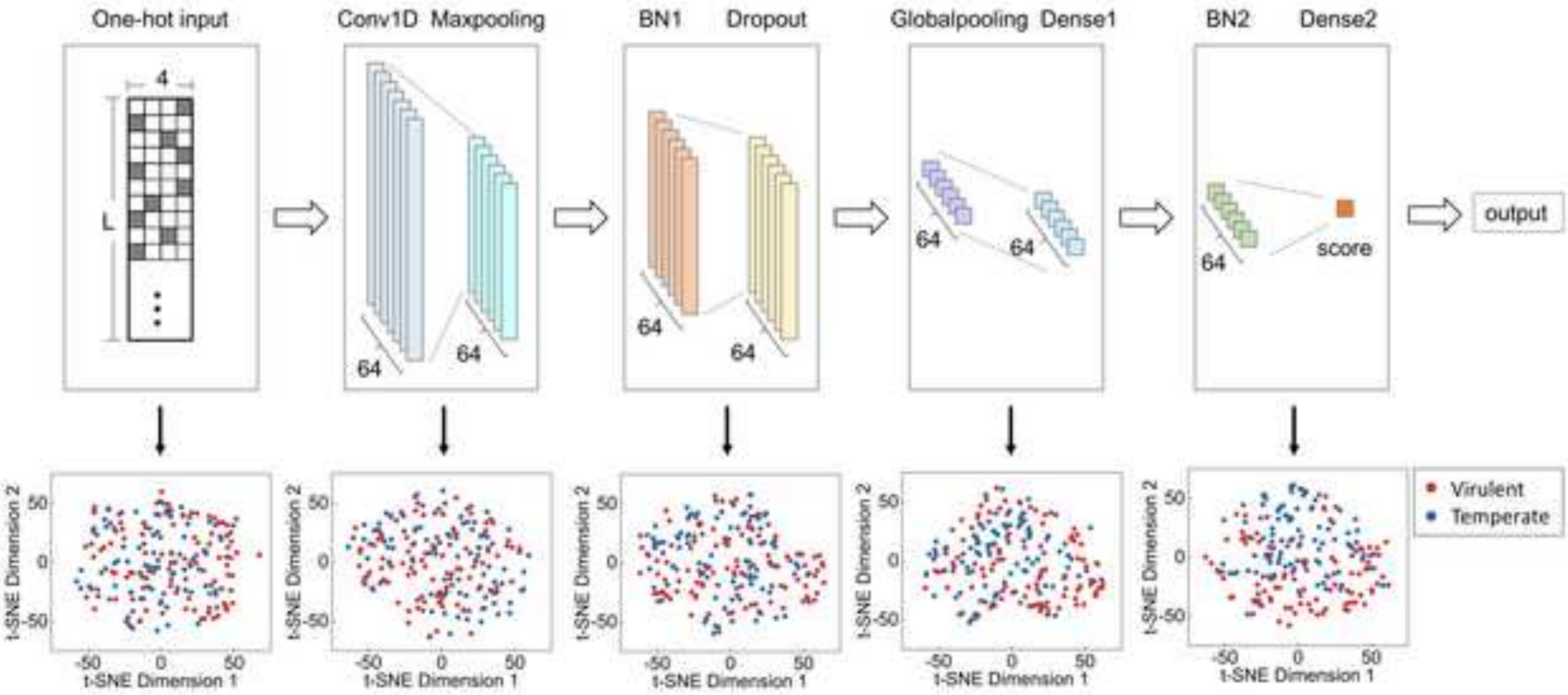

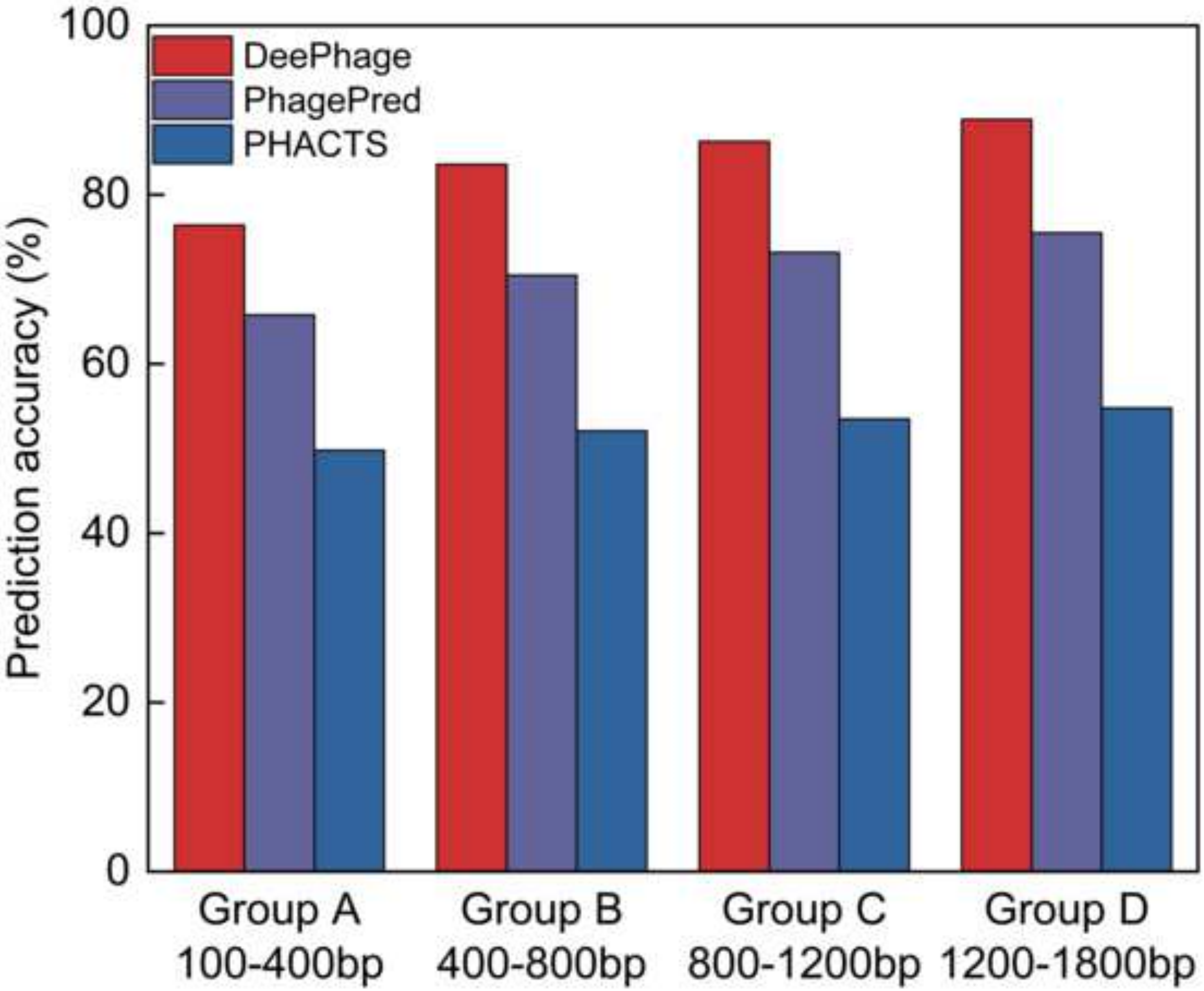
100
80
60
40
20
0
Prediction accuracy (%)
DeePhage
PhagePred
PHACTS
Group A
100-400bp
Group B
400-800bp
Group C
800-1200bp
Group D
1200-1800bp

Figure_3_revised Click here to access/download;Figure;Figure_3_revised.png

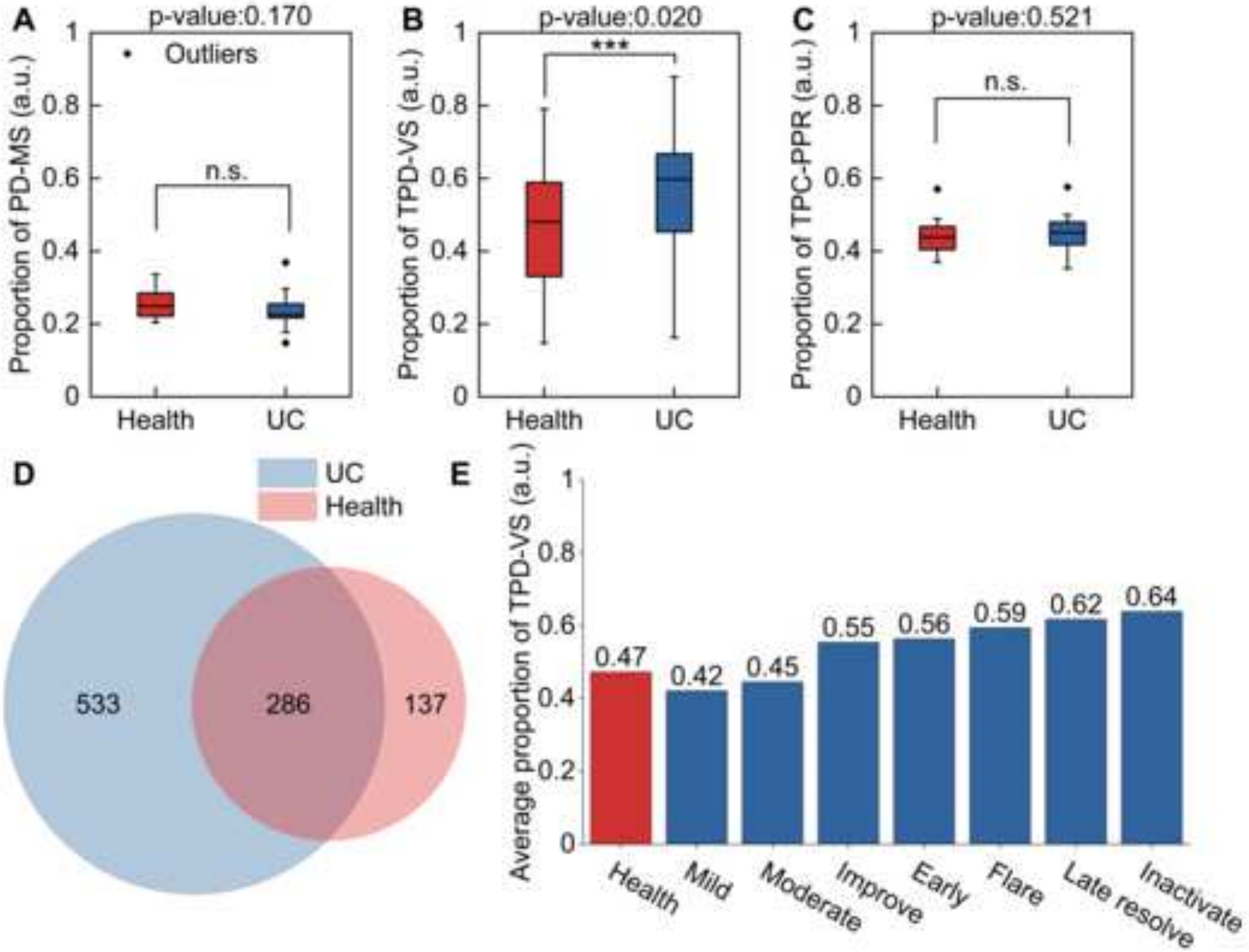

Additional File 1

Click here to access/download
Supplementary Material
Additional_File_1_0709.docx

Additional File 2

Click here to access/download
Supplementary Material
Additional_File_2_0709.xlsx

Cover_letter

Click here to access/download
Supplementary Material
Cover_Letter_R2_revised.docx
