# Supplementary material for: DeePhage: distinguishing virulent and temperate phage-derived sequences in metavirome data with a deep learning approach
Source: Gigascience. 2021 Sep 8;10(9):giab056. doi: 10.1093/gigascience/giab056 (PMC8427542; doi:10.1093/gigascience/giab056)
Supplement: giab056_Response_to_Reviewer_Comments_Original_Submission [file giab056_response_to_reviewer_comments_original_submission.pdf]

Author's Response To Reviewer Comments

Close

Dear Editor,

Thank you very much for your previous E-mail on February 12, 2021 regarding our manuscript ‘‘Distinguish virulent and temperate phage-derived sequences in metavirome data with a deep learning approach’’ (Manuscript ID: GIGA-D-20-00378). We are very grateful to your substantial and helpful advices on the manuscript. We thank so many substantial and valuable advices from two reviewers, including their careful reading and check to the manuscript, which clearly helped us improve the paper. Following your instructions and the reviewers’ comments, we have made a conscious effort to revise the manuscript (both the main document and Additional File) with an essential improvement.
Since submitting system are not allowed us to upload a single file of cover letter, we upload our full cover letter in the form of Supplementary Material, which is called "Cover Letter". Please refer to that Supplementary Material to see our response to Editor and two reviewers.
We hope that revision will clarify all the points by two reviewers and give a point-by-point response to all the concerns, we hereby resubmit our manuscript to GigaScience. We thank you for your kind consideration.

Sincerely yours,
Huaiqiu Zhu, Ph. D., Professor
Peking University

Close
