# Supplementary material for: DeePhage: distinguishing virulent and temperate phage-derived sequences in metavirome data with a deep learning approach
Source: Gigascience. 2021 Sep 8;10(9):giab056. doi: 10.1093/gigascience/giab056 (PMC8427542; doi:10.1093/gigascience/giab056)
Supplement: giab056_Reviewer_1_Report_Original_Submission — Satoshi Hiraoka -- 1/25/2021 Reviewed [file giab056_reviewer_1_report_original_submission.pdf]

Reviewer Report

Title: DeePhage: distinguish virulent and temperate phage-derived sequences in metavirome data with a deep learning approach

Version: Original Submission **Date:** 1/25/2021

Reviewer name: Satoshi Hiraoka

Reviewer Comments to Author:

In this manuscript, Wu et al. developed a novel bioinformatics tool, DeePhage, for classifying viral genomes into virulent and temperate phage types in metagenomic settings. The classification is based on a deep learning method and showed better performance (i.e., accuracy and calculation speed) than PHACTS, which was a similar tool but focuses on near-complete viral genome and useless for metagenomic sequencing reads. In addition, the DeePhage tool was applied to real metagenomic and metaviromic dataset resulted in several interesting findings

The manuscript is overall well written. The purpose of this study is also important because the viral lifecycle is amost always overlooked in genomic analysis of environmental microbes and viruses. While I'm not a specialist in machine learning, I think the developed method seems to be typical and reasonable. In addition to the manuscript, online manual and GitHub page were also well prepared that could facilitate easy usage for researchers including bioinformatics beginners. Although the further improvement of performance and evaluation of the analysis should be required in further study, mainly due to the insufficient teaching dataset currently available, the novel tool could be a trigger to shed light on the viral ecology in nature, and thus the study could have a potential impact on wide fields of microbiology and virology.

I have several suggestions, both major and minor, that if addressed would increase the clarity and impact of this manuscript.

Major comment:

In this study, a total of 225 annotated viral genomes were used for teaching data. One of my concerns is that the teaching data size is likely still insufficient for reliable classification. And also I concerned whether the dataset may compose unevenly-selected genomes, for example, were some specific viral lineages unevenly abundant in the dataset? The author should provide list of the used viral genomes (maybe as same as the McNair dataset) with several descriptions and statistics if possible (e.g., host linage, viral lineage, genome size, etc.) in this paper or GitHub site. This not only provides details of the analysis but also allow someone for further maintenance, improvement, and expansion of the teacher dataset. Because the kinds of database biases should affect downstream analysis and method evaluation as similar to the unevenness of positive/negative samples (as described in L136-143), it would be better to add more explanations of the potential database biases and limitations of the classification accuracy/sensitivity when using current teacher dataset in this manuscript.

L315-320. I think this cutoff is useful in many cases in real usage of actual data analysis because 'reliable' results are more valuable in various scientific studies rather than those mixed with uncertain classification. Could the author provide a recommended value of the cutoff and set this as a default

option of the tool? Why the 'uncertain' label was not used in the downstream analysis in this study? Besides, I'm also interested in the distribution of the score when applying the tool to each dataset: artificial virome, rumen virome, human gut metagenome, and human gut virome. It may be expected bipolar distribution (i.e., ~1 or ~0) when considering bar plot of score vs. number of contigs, right? In contrast, I presumed the analysis of RefSeq bacterial genomes (L521-543) showed scores most of them were close to 0.5 because the bacterial reads will not appropriately be classified in each of the virulent/temperate types with higher reliability than actual viral contigs. Is this correct?
Minor comment:
Title. Because the main result of this study was the development of the tool, I think it would be better to add the term 'DeePhage' into the title to clarify this.
L41. add citation. ([1]?)
L44. What ' untargeted' mean? Is this extra term necessary in this manuscript?
L68-69. Some prophages are known as 'plasmid prophage' which are not integrated into host chromosome but independently state like plasmid in host cell. (e.g., https://www.sciencedirect.com/science/article/pii/S0042682217304245)
L162-165. 'they have different sequence signature' here, but it seems to be a bit hard to separate virulent/temperate phage genomes in Figure 1. PCA analysis frequently hard to visualize clear separations in categories, and this statement is likely misreading. Hence I recommend that figure 1 will move to supplemental information and remove later text of this sentence (', showing that ~ phage genomes' in L164-165).
L352-359. Was this performance measured using one CPU thread? This is a comment and not required for this manuscript, but I think an additional option of parallel calculation with multiple threads should be much convenient for most users because typical metagenomic/virome datasets contain numbers of sequencing reads that take the great computational cost for those analyses.
L408. The comparative analysis of human gut metagenome and virome dataset is complicated. Additional figure of the analysis scheme and/or summarized table of the results of these analyses should greatly improve readability.
L533. How to chose the 120 genomes from RefSeq? Random selection?
Figure 3. Why the y-axis was started from 40% instead of 0%? This could give a false impression to readers.

Methods

Are the methods appropriate to the aims of the study, are they well described, and are necessary controls included? Choose an item.

Conclusions

Are the conclusions adequately supported by the data shown? Choose an item.

Reporting Standards

Does the manuscript adhere to the journal's guidelines on [minimum standards of reporting?] Choose an item.

Choose an item.

Statistics

Are you able to assess all statistics in the manuscript, including the appropriateness of statistical tests used? Choose an item.

Quality of Written English

Please indicate the quality of language in the manuscript: Choose an item.

Declaration of Competing Interests

Please complete a declaration of competing interests, considering the following questions:

- Have you in the past five years received reimbursements, fees, funding, or salary from an organisation that may in any way gain or lose financially from the publication of this manuscript, either now or in the future?
- Do you hold any stocks or shares in an organisation that may in any way gain or lose financially from the publication of this manuscript, either now or in the future?
- Do you hold or are you currently applying for any patents relating to the content of the manuscript?
- Have you received reimbursements, fees, funding, or salary from an organization that holds or has applied for patents relating to the content of the manuscript?
- Do you have any other financial competing interests?
- Do you have any non-financial competing interests in relation to this paper?

If you can answer no to all of the above, write 'I declare that I have no competing interests' below. If your reply is yes to any, please give details below.

I declare that I have no competing interests

I agree to the open peer review policy of the journal. I understand that my name will be included on my report to the authors and, if the manuscript is accepted for publication, my named report including any attachments I upload will be posted on the website along with the authors' responses. I agree for my report to be made available under an Open Access Creative Commons CC-BY license (http://creativecommons.org/licenses/by/4.0/). I understand that any comments which I do not wish to be included in my named report can be included as confidential comments to the editors, which will not be published.

Choose an item.

To further support our reviewers, we have joined with Publons, where you can gain additional credit to further highlight your hard work (see: https://publons.com/journal/530/gigascience). On publication of this paper, your review will be automatically added to Publons, you can then choose whether or not to claim your Publons credit. I understand this statement.

Yes Choose an item.
