# Supplementary material for: DeePhage: distinguishing virulent and temperate phage-derived sequences in metavirome data with a deep learning approach
Source: Gigascience. 2021 Sep 8;10(9):giab056. doi: 10.1093/gigascience/giab056 (PMC8427542; doi:10.1093/gigascience/giab056)
Supplement: giab056_Reviewer_2_Report_Original_Submission — Sidi Chen -- 2/11/2021 Reviewed [file giab056_reviewer_2_report_original_submission.pdf]

Reviewer Report

Title: DeePhage: distinguish virulent and temperate phage-derived sequences in metavirome data with a deep learning approach

Version: Original Submission **Date:** 2/11/2021

Reviewer name: Sidi Chen

Reviewer Comments to Author:

Wu et al. presents a computational tool for identifying virome sequences as virulent or temperate phage-derived fragments. They construct artificial contigs extracted from 127 complete phage genomes with lifestyle annotations, and built a framework integrating one-hot encoding and convolutional neural networks for a binary classification task. The authors tested six different architectures varying parameters such as encoding representations and removing certain layers on different groups of artificial contigs (based on varying lengths) to find that their DeePhage architecture had the best overall performance. The authors then used their methods to predict sequences in metavirome data of bovine rumen and from an ulcerative colitis (UC) study as applications of real metavirome data.Throughout the manuscript the authors use the previously published tool Phage Classification Tool Set (PHACTS) as a point of comparison, but in fact uses the same dataset from for addressing essentially the same problem. The novelty proposed by the authors here appears to be that DeePhage is a read or contig based approach which is adapted for metagenomic fragments.

(1) While each individual step is acceptable, there is a high degree of abstraction and redundancy involved in the study, including using artificial contigs (are there real world annotated virome datasets that can be used as inputs?) and the same dataset as the PHACTS (are there any other high confidence annotations?). The authors should systematically review dataset construction strategies and test different input setups where possible.

(2) The authors used PCA on 4-mer frequencies (Figure 1) to show differences in sequence signatures, but the variance captures by the PCs is not striking and this approach is also not statistically rigorous. On top of that, 4-mer frequencies are not used as an input to the model. The authors should perform statistical analyses on the input data to their ML model to show significant differences.

(3) When comparing PHACTS and DeePhage on metavirome data of bovine rumen, the authors generated a mini temperate phage-derived protein set to generate a surrogate set of "temperate" contigs. However, only 16/118918 contigs had homology. The authors should test other real world datasets to see if this is a general feature of metavirome data or just characteristic of bovine rumen. Furthermore, they should test their tools on the contigs without homology to see the false positive rates.

(4) The authors analyze virome data from ulcerative colitis (UC) patients and healthy people to find associations between phages and gut microbiota, but should describe the utility and biological correlates of their analyses more clearly and also ideally demonstrate them with another real world dataset.

(5) Can the authors use DeePhage to make inferences in a species-aware manner?

(6) Are there any sequence-based features learned throughout the training process (not just separation of labels)?
(7) Minor - the manuscript could use some proof-reading throughout, e.g. "following" to "followed" on line 71, "making" to "make" on line 75.

Methods

Are the methods appropriate to the aims of the study, are they well described, and are necessary controls included? Choose an item.

Conclusions

Are the conclusions adequately supported by the data shown? Choose an item.

Reporting Standards

Does the manuscript adhere to the journal's guidelines on [minimum standards of reporting?] Choose an item.

Choose an item.

Statistics

Are you able to assess all statistics in the manuscript, including the appropriateness of statistical tests used? Choose an item.

Quality of Written English

Please indicate the quality of language in the manuscript: Choose an item.

Declaration of Competing Interests

Please complete a declaration of competing interests, considering the following questions:

- Have you in the past five years received reimbursements, fees, funding, or salary from an organisation that may in any way gain or lose financially from the publication of this manuscript, either now or in the future?
- Do you hold any stocks or shares in an organisation that may in any way gain or lose financially from the publication of this manuscript, either now or in the future?
- Do you hold or are you currently applying for any patents relating to the content of the manuscript?
- Have you received reimbursements, fees, funding, or salary from an organization that holds or has applied for patents relating to the content of the manuscript?
- Do you have any other financial competing interests?

- Do you have any non-financial competing interests in relation to this paper?

If you can answer no to all of the above, write 'I declare that I have no competing interests' below. If your reply is yes to any, please give details below.

N/A

I agree to the open peer review policy of the journal. I understand that my name will be included on my report to the authors and, if the manuscript is accepted for publication, my named report including any attachments I upload will be posted on the website along with the authors' responses. I agree for my report to be made available under an Open Access Creative Commons CC-BY license (http://creativecommons.org/licenses/by/4.0/). I understand that any comments which I do not wish to be included in my named report can be included as confidential comments to the editors, which will not be published.

Choose an item.

To further support our reviewers, we have joined with Publons, where you can gain additional credit to further highlight your hard work (see: https://publons.com/journal/530/gigascience). On publication of this paper, your review will be automatically added to Publons, you can then choose whether or not to claim your Publons credit. I understand this statement.

Yes Choose an item.
