# Supplementary material for: DeePhage: distinguishing virulent and temperate phage-derived sequences in metavirome data with a deep learning approach
Source: Gigascience. 2021 Sep 8;10(9):giab056. doi: 10.1093/gigascience/giab056 (PMC8427542; doi:10.1093/gigascience/giab056)
Supplement: giab056_Reviewer_2_Report_Revision_1 — Sidi Chen -- 6/25/2021 Reviewed [file giab056_reviewer_2_report_revision_1.pdf]

Reviewer Report

Title: DeePhage: distinguish virulent and temperate phage-derived sequences in metavirome data with a deep learning approach

Version: Revision 1 **Date:** 6/25/2021

Reviewer name: Sidi Chen

Reviewer Comments to Author:

Overall, the authors have developed a deep learning based tool for classifying phage genomes fragments as a virulent or temperate, with an expanded dataset and several new analyses in this revision. The authors also perform additional analysis.
First, there is no point-by-point response to previous round of reviewer's comments, which is essential. From reading of R1 itself it looks like the authors made efforts to address previous comments, however it's not elaborated in response.
While the tool may be useful, there are still some reservations regarding the quality of the data both for training and downstream analyses purposes. The reviewer understand that some of the limitations are due to the limitations in the data currently available.
Specific comments below:
(1) The authors discuss the difference between DeePhage and PHACTs, emphasizing the focus of their tool to be based on metagenomic fragments. They also introduce discussions about and comparisons to the new tool PhagePred. The reviewer acknowledge the value of developing a classifier for phage lifestyle based on metagenomic data, but the main concern was that the dataset (e.g. phage genomes and lifestyle annotations) overlapped exactly with the McNair dataset. To this point, the authors curated another dataset (termed "Dataset-2") that included more phage RefSeq genomes from the NCBI database. The authors write that they "used all Dataset-2 and four fifths of Dataset-1 as the training set, and one fifth of Dataset-1 as the test set" - how was the input dataset partitioned? Were the test set selected randomly? Were they selected by genome or by short sequences? Notably, "Additional File 2" do not have genomes labelled by whether they were used for test or training class. If they used different partitions for cross-validations and the full dataset for final model, this should be stated explicitly. In fact, it would be helpful if the authors provide figures detailing basic statistics (e.g. labels, partitions, lineages, etc.) on their datasets.
(2) Considering both the significant difference in size and nature of Dataset-1 and 2, and therefore the initial pipeline and the revised one, the authors should compare (at least internally) and clarify which dataset was used for which analyses. Notably: Have the entire pipeline, subsequent analyses (e.g. PCAs), and github implementations described in the main text been updated with combined dataset? How does the performance change between Dataset-1 only and the combined dataset?
(3) The authors write "We have tested that the traditional k-mer frequency encoding form was not superior to the one-hot encoding form." - can they elaborate on this?
(4) The authors find differential category distribution according to the COG database for proteins predicted for virulent or temperate phages - the authors should contextualize their findings with what is

known in the literature.

Methods

Are the methods appropriate to the aims of the study, are they well described, and are necessary controls included? Choose an item.

Conclusions

Are the conclusions adequately supported by the data shown? Choose an item.

Reporting Standards

Does the manuscript adhere to the journal's guidelines on minimum standards of reporting? Choose an item.

Choose an item.

Statistics

Are you able to assess all statistics in the manuscript, including the appropriateness of statistical tests used? Choose an item.

Quality of Written English

Please indicate the quality of language in the manuscript: Choose an item.

Declaration of Competing Interests

Please complete a declaration of competing interests, considering the following questions:

- Have you in the past five years received reimbursements, fees, funding, or salary from an organisation that may in any way gain or lose financially from the publication of this manuscript, either now or in the future?
- Do you hold any stocks or shares in an organisation that may in any way gain or lose financially from the publication of this manuscript, either now or in the future?
- Do you hold or are you currently applying for any patents relating to the content of the manuscript?
- Have you received reimbursements, fees, funding, or salary from an organization that holds or has applied for patents relating to the content of the manuscript?
- Do you have any other financial competing interests?
- Do you have any non-financial competing interests in relation to this paper?

If you can answer no to all of the above, write 'I declare that I have no competing interests' below. If your reply is yes to any, please give details below.

'I declare that I have no competing interests'

I agree to the open peer review policy of the journal. I understand that my name will be included on my report to the authors and, if the manuscript is accepted for publication, my named report including any attachments I upload will be posted on the website along with the authors' responses. I agree for my report to be made available under an Open Access Creative Commons CC-BY license (http://creativecommons.org/licenses/by/4.0/). I understand that any comments which I do not wish to be included in my named report can be included as confidential comments to the editors, which will not be published.

Choose an item.

To further support our reviewers, we have joined with Publons, where you can gain additional credit to further highlight your hard work (see: https://publons.com/journal/530/gigascience). On publication of this paper, your review will be automatically added to Publons, you can then choose whether or not to claim your Publons credit. I understand this statement.

Yes Choose an item.
