# Supplementary material for: DeePhage: distinguishing virulent and temperate phage-derived sequences in metavirome data with a deep learning approach
Source: Gigascience. 2021 Sep 8;10(9):giab056. doi: 10.1093/gigascience/giab056 (PMC8427542; doi:10.1093/gigascience/giab056)
Supplement: giab056_Supplemental_Files [file giab056_supplemental_files.zip › Cover_Letter_R2_revised.docx]

**Cover Letter**

Dear Editor,

Thank you very much for your previous E-mail on June 28, 2021 regarding our revised manuscript “DeePhage: distinguish virulent and temperate phage-derived sequences in metavirome data with a deep learning approach” (Manuscript ID: GIGA-D-20-00378R1). With our last revision, we are pleased to know that our responses satisfied both of the reviewers as “the manuscript was much improved from the last one” and “…with an expanded dataset and several new analyses in this revision”. Also, we are very glad to hear from the editor that our manuscript is potentially acceptable for publication in this journal. Additionally, we would like to thank two reviewers for their careful reading for both original and the revised manuscript. The comments raised by two reviewers helped us make further improvements.

Following your instruction and Reviewers’ comments, this time we have made a conscious effort to revise the manuscript with further improvement. As a respond to the editor’s instructions, actually we have registered DeePhage in the SciCrunch.org database since the first submission. In the section of “Availability of supporting source code and requirements”, we have provided the RRID (SCR_019243) (Please refer to Line 621, Page 22 in the revised manuscript). In addition, we noted that the PhagePred tool has been published on November, 2020 nearly at the same time of first submitting our manuscript on December, 2020. So, we decided to change the claim that our Deephage is the first tool for phage lifestyle classification for viral metiagenomic data. Even though, DeePhage belongs to the state-of-the-art tools. In the last round review response, we have fully discussed and compared with the related tools. To keep the preciseness of our manuscript, we changed the words “To the best of our knowledge, DeePhage is the first tool to” as “DeePhage is a novel tool developed to”, changed the words “to the best of our knowledge, DeePhage is the first tool that” as “DeePhage is a novel tool that” in the revised manuscript. (Please refer to Line 30, Page 2 and Line 601, Page 21 in the revised manuscript.)

In the revised manuscript, all the changed words, sentences and paragraphs are marked in red text. We then report our revisions and responses to two reviewers’ all comments (italic text) one by one as follows:

***To Reviewer 1:***

**General Comments:** *The manuscript was much improved from the last one. The response to the reviewer's concerns has greatly strengthened the manuscript. I have a few additional (unimportant) recommendations as follows.*

We thank Reviewer 1 for his/her careful reading and efforts to improve our manuscript. It’s glad to see that our previous responses were adequately addressed all the points raised by Reviewer 1. The positive comment that “*The manuscript was much improved from the last one. The response to the reviewer's concerns has greatly strengthened the manuscript*” is really encouraging. The remaining comments raised by Reviewer 1 would help a lot for improving the quality of the manuscript. Below, we itemize our revisions in response to Reviewer 1’s points.

**Minor comments:**

**Minor comment 1:** *Table 2. Hits length -> Hit length (bp)*

We thank Reviewer 1 for this comment. We have changed “Hits length” to “Hit length (bp)” in Table 2 in the revised manuscript. (Please refer to Line 419, Page 15 in the revised manuscript.)

**Minor comment 2:** *Figure S1. Check upper/lower cases in labels of x- and y-axes. "First Principal Component" -> "First principal component", and "Second Principal Component" -> "Second principal component"*

We thank Reviewer 1 for noting the typos in Figure S1. In Figure S1, we have corrected “First Principal Component” to “First principal component” and “Second Principal Component” to “Second principal component”. (Please refer to Figure S1 in the revised Additional File 1.)

**Minor comment 3:** *Additional file 2. Taxonomic columns would be sorted in decreasing order (i.e., Kingdom, Phylum, Class, Order, Family, Genus, Species).*

We thank Reviewer 1 for reminding us to sort the taxonomic columns in decreasing order. In the revised Additional File 2, we have sorted all the phage and its host taxonomic columns in decreasing order. (Please refer to the revised Additional File 2.)

***To Reviewer 2:***

**General Comments:** *Overall, the authors have developed a deep learning based tool for classifying phage genomes fragments as a virulent or temperate, with an expanded dataset and several new analyses in this revision. The authors also perform additional analysis.*

*First, there is no point-by-point response to previous round of reviewer's comments, which is essential**. From reading of R1 itself it looks like the authors made efforts to address previous comments, however it's not elaborated in response.*

*While the tool may be useful, there are still some reservations regarding the quality of the data both for training and downstream analyses purposes. The reviewer understand that some of the limitations are due to the limitations in the data currently available.*

We thank Reviewer 2 for the comments to the last round review response. We are pleased to receive the positive judgement as ‘*Overall, the authors have developed a deep learning based tool for classifying phage genomes fragments as a virulent or temperate, with an expanded dataset and several new analyses in this revision*’. Also, we appreciate that Reviewer 2 understood some of our limitations are owing to the data limitations. We believe that such limitations will be improved by the future development of high-quality data. In addition, we have tried our best to present our answers to Reviewer 2’s all concerns point-by-point in the last revision, while Reviewer 2 mentioned that ‘*there is no point-by-point response to previous round of reviewer's comments*’, and Editor has noticed that there might be some misunderstandings and had emailed Reviewer 2 with our last response separately. Herein we thank both Editor and Reviewer 2 for their understanding our work seriously and responsibly. We then report our responses to Reviewer’s further comments as follows.

**Specific comments1:** *The authors discuss the difference between DeePhage and PHACTs, emphasizing the focus of their tool to be based on metagenomic fragments. They also introduce discussions about and comparisons to the new tool PhagePred. The reviewer acknowledge the value of developing a classifier for phage lifestyle based on metagenomic data, but the main concern was that the dataset (e.g. phage genomes and lifestyle annotations) overlapped exactly with the McNair dataset. To this point, the authors curated another dataset (termed "Dataset-2") that included more phage RefSeq genomes from the NCBI database. The authors write that they "used all Dataset-2 and four fifths of Dataset-1 as the training set, and one fifth of Dataset-1 as the test set" - how was the input dataset partitioned? Were the test set selected randomly? Were they selected by genome or by short sequences? Notably, "Additional File 2" do not have genomes labelled by whether they were used for test or training class. If they used different partitions for cross-validations and the full dataset for final model, this should be stated explicitly. In fact, it would be helpful if the authors provide figures detailing basic statistics (e.g. labels, partitions, lineages, etc.) on their datasets.*

We are glad to hear that Reviewer 2 admit the value of our work, which is different from PHACTS as we have denoted in the last round review response. However, Reviewer 2 was confused about the usage of new dataset (Dataset-2), we realize that we have not clarified this important point. Therefore, we would like to describe our detailed data processing process point-by-point.

(1) “*how was the input dataset partitioned?*”, “*Were the test set selected randomly? Were they selected by genome or by short sequences?*”

All Dataset-2 and four fifths of Dataset-1 were used as training set and one-fifth of Dataset-1 were used as test set. In detail, the partition of Dataset-1 for training and test is randomly selected by genome. We did a five-fold cross validation as follows. Dataset-1 (225 phage genomes) was randomly separated into five parts. Each part was then used as test set in each cross validation and the rest were used as the training set. Statistically, there are nearly 1820 (180 from four-fifth Dataset-1 and 1640 from all Dataset-2) phage genomes for training and 45 (one-fifth Dataset-1) phage genomes for test in each rotation. Thus, each phage genome in Dataset-1 is used as the test set one time only. Then the MetaSim software was used to simulate 80000 and 20000 short fragments for training and test. To clarify this point, we revised the sentence “Therefore, we used all phages of Dataset-2 and four-fifth phages of Dataset-1 to be the training set, and one-fifth phages of Dataset-1 to be the test set.” to “Therefore, based on genomes, we used all phages of Dataset-2 and four-fifth phages of Dataset-1 to be the training set, and one-fifth phages of Dataset-1 to be the test set.” (Please refer to Line 146-148, Page 5 in the revised manuscript.). More information about the data processing is described in the manuscript as “To test whether DeePhage can distinguish the lifestyle of novel phages or not, for each validation, we divided the training set and the test set based on complete genomes rather than artificial contigs, and then simulated 80,000 training sequences and 20,000 test ones using MetaSim [26].” (Please refer to Line 293-296, Page 11 in the revised manuscript.)

(2) “*Additional File 2’ do not have genomes labelled by whether they were used for test or training class*.”

To be clearer and in accordance with the Reviewer’s concerns, we added the usage label (Training usage or Test usage) of each phage genomes in Additional File 2. Usage labels indicate in which cross validation the phage genome was used for training or test set. We added the sentence “Moreover, a usage label (Training usage or Test usage) was used to indicate in which cross validation the phage genome was used for training or test set in Additional File 2.” (Please refer to Line 149-151, Page 6 in the revised manuscript.) Also, we added the column of usage label for each phage genomes in Additional File 2. (Please refer to the revised Additional File 2.)

(3) “*it would be helpful if the authors provide figures detailing basic statistics (e.g. labels, partitions, lineages, etc.) on their datasets.*”

Labels and partitions information has been added into Additional File 2 according to items (1) and (2). As for the information of lineages, our previous manuscript has included the lineages information of each phage genomes in Additional File 2. Also, we have sorted all the phage and its host taxonomic columns in decreasing order following the suggestion of Reviewer 1. (Please refer to the revised Additional File 2.)

**Specific comments2:** *Considering both the significant difference in size and nature of Dataset-1 and 2, and therefore the initial pipeline and the revised one, the authors should compare (at least internally) and clarify which dataset was used for which analyses. Notably: Have the entire pipeline, subsequent analyses (e.g. PCAs), and github implementations described in the main text been updated with combined dataset? How does the performance change between Dataset-1 only and the combined dataset?*

The PCA analysis did not update, since we still used phage genomes only from Dataset-1. To be clearer, we revised the sentence “…, among virulent phage genomes and temperate phage genomes” to “…, among virulent phage genomes and temperate phage genomes from Dataset-1.” (Please refer to Line 168-169, Page 6 in the revised manuscript.)

Internally, DeePhage’s performance improved on five-fold cross validation set after using the combined dataset, compared with using only Dataset-1. In each length group, the Acc criterion increased 1~2%. It means dataset expanding is useful. For the remain analysis, the performance of DeePhage using the combined dataset slightly altered and have no effects on any conclusion. Thus, we updated all the results using combined dataset in the current manuscript, except for the PCA analysis. Those updated results include analysis on five-fold cross validation, all phage CDS sequences, metavirome from bovine rumen dataset, metagenome/metavirome from healthy/ulcerative-colitis-patients and so on. Those changes were also reported in the response to Reviewer 2’s comment #1 in the last round review response.

**Specific comments3:** *The authors write "We have tested that the traditional k-mer frequency encoding form was not superior to the one-hot encoding form." - can they elaborate on this?*

As shown in Figure S2 of the previous Additional File 1, we designed a Kmer-4 model, which used k-mer frequencies as an encoding representation, and the architecture of Kmer-4 model was similar to that of DeePhage except to the input layer. Also, we presented that the Kmer-4 model did a terrible prediction as shown in Table S1 of the previous Additional File 1.

Thus, to be clearer, we revised the sentence “We have tested that the traditional k-mer frequency encoding form was not superior to the one-hot encoding form.” to “We have tested that the traditional k-mer frequency encoding form was not superior to the one-hot encoding form, since Kmer-4 model did a terrible prediction as shown in Additional File 1 (Table S1).” (Please refer to Line 536-537, Page 19 in the revised manuscript.)

**Specific comments4:** *The authors find differential category distribution according to the COG database for proteins predicted for virulent or temperate phages - the authors should contextualize their findings with what is known in the literature.*

We thank Reviewer 2 for this suggestion. As we can see in Figure S4, the biggest difference between virulent and temperate protein sequences is located at ‘Mobilome: prophages, transposons’ category”. Temperate phages could exist as prophages and mediate horizontal gene transfer via transduction, while such genetic exchanges might be rare when involving virulent phages. Thus, such the difference could be comprehended and obviously learned by DeePhage. We added the sentence “As we can see in Figure S4, the biggest difference between virulent and temperate protein sequences is located at ‘Mobilome: prophages, transposons’ category. Temperate phages could exist as prophages and mediate horizontal gene transfer via transduction [51], while such genetic exchanges might be rare when involving virulent phages [52]. Thus, such difference could be comprehended and obviously learned by DeePhage.” in the revised manuscript (Please refer to Line 584-588, Page 21 in the revised manuscript.)

In hoping that the above revision has clarified all the points by two reviewers and given a point-by-point response to all the concerns, we hereby resubmit our manuscript to the journal. We thank you for your kind consideration.

Sincerely yours,

Huaiqiu Zhu, Ph. D., Professor

Peking University
